# Bioengineered silicon nanoparticles in agroecosystems: molecular mechanisms, stress tolerance, and environmental implications

**DOI:** 10.3389/fpls.2026.1814785

**Published:** 2026-07-16

**Authors:** Swati Sachdev, Syed Uzma Jalil, Zienab F. R. Ahmed, Mohammad Israil Ansari

**Affiliations:** 1 Department of Liberal Education, Era University, Lucknow, India; 2 Amity Institutes of Biotechnology, Amity University, Lucknow, India; 3 Department of Integrative Agriculture, College of Agriculture and Veterinary Medicine, United Arab Emirates University, Al Ain, United Arab Emirates; 4 Zayed Center for Health Sciences, United Arab Emirates University, Al Ain, United Arab Emirates; 5 Department of Botany, University of Lucknow, Lucknow, India

**Keywords:** antioxidant defense system, nutrient uptake, oxidative damage, signaling pathway, silicon transporters

## Abstract

The increasing incorporation of engineered nanomaterials into agro-environmental systems requires a balanced evaluation of their biological efficacy and potential environmental implications. Silicon (Si) is a quasi-essential element, which is not only essential for plant survival but also supports their growth under adverse conditions. The large size and tendency to form complexes with inorganic and organic compounds in the growth medium, the bioavailability of Si to plants is limited. These restrictions can be overwhelmed through the application of silicon nanoparticles (SiNPs), which typically range from 1 to 100 nm and exhibit better mobility, solubility, and bioavailability. SiNPs can be synthesized by physical, chemical, and biological methods; however, the biological approach has garnered appreciable response due to multiple ecological benefits, biocompatibility, reduced energy requirements, and cost-effectiveness. Biosynthesized SiNPs are taken up by the plants as silicic acid via transporters viz., Ls1, Ls2, and Ls6, and/or by endocytosis depending upon their size, physicochemical properties, and plant species. Following internalization, SiNPs are predominantly transported to the epidermal cell wall, where it forms silica-cuticle double layer, providing resistance to biotic and abiotic stresses. Accumulation of SiNPs improves plants’ water use efficiency, nutrient uptake and assimilation, photosynthetic performance, and antioxidant-based defense system, thereby stimulating plant growth and productivity under normal and stressed conditions such as drought, heat, salinity, and heavy metals stress. At molecular level, SiNPs demonstrated activation of signaling pathways by inducing transient and tightly regulated reactive oxygen species (ROS) burst, which activates phytohormones and MAPK signaling cascade, eventually triggering expression of stress-responsive genes. Biosynthesized SiNPs hold immense potential in field of agriculture, however, their actual deployment is at infancy. A lack of comprehensive understanding of SiNP mediated stress alleviation in plants at molecular level coupled with variability in response, subjected to plant species, and morphological and physicochemical attributes of SiNPs, limits their large-scale application. Thus, elucidating precise molecular mechanism of biosynthesized SiNPs-mediated stress tolerance in plants is crucial. Moreover, integrating advanced biological approaches with emerging technologies can facilitate rational and sustainable implementation of nano-enabled agricultural practices.

## Graphical Abstract

**Figure:**
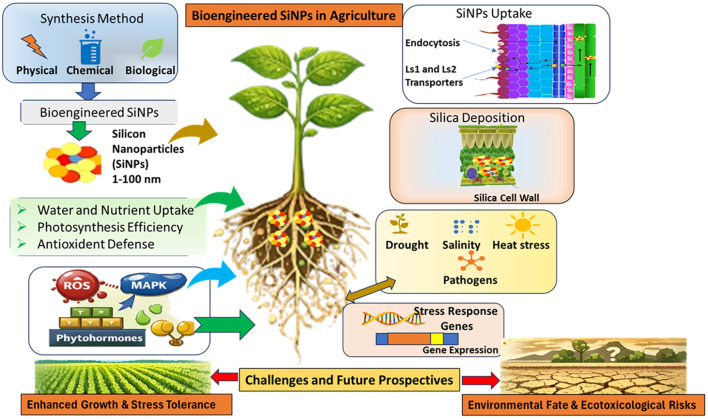

## 1 Introduction

The extensive reliance on conventional agricultural activities to feed a thriving global population, coupled with gradual climate change, has increased the pressure of biotic and abiotic stresses on agro-ecosystems. Plants growing under the influence of environmental stresses such as heat, water-deficient conditions, poor nutrient availability, salinity, presence of toxic metals, and pests and pathogen infestation are characterized by poor development and low productivity due to the induced physiological, biochemical, and molecular alterations, which hinder water and nutrient uptake, nutrient assimilation, and the photosynthetic process (Costa et al., 2024; Antu et al., 2025). Exposure to unfavorable conditions triggers overproduction of reactive oxygen species (ROS), exceeding the plant’s ability to manage them, which disrupts cell membranes and damages biomolecules, affecting photosynthesis and other major processes (Sachdev et al., 2021, Sachdev et al., 2023). Beyond crop yield losses, stress-induced damage also influences soil health, biogeochemical cycling, and the stability of agro-ecosystems, thereby raising broader environmental and food-security concerns. To address the burgeoning issue, several advances have been put forward, including the application of nanoparticles. The nanoparticles are always more considerable than their bulk counterpart due to their extraordinary physicochemical properties, such as high solubility and reactivity, attributed to a large surface area-to-volume ratio and small size (Sachdev and Ahmad, 2021; Bhat et al., 2021). Nanoparticles (NPs) are effective at low dosages; hence, their use can minimize the input of synthetic chemicals, alleviating the risk of environmental pollution (Jalil and Ansari, 2021; Miao et al., 2025). NPs are synthesized in various morphologies, including spherical, rod-shaped, mesoporous, and hollow, with diameters typically ranging from 1 to 100 nm (Jalil and Ansari, 2019; Naaz et al., 2023; Yan et al., 2024) through physical, chemical, and biological (green synthesis) methods. The bioengineering approach for synthesis has garnered global interest, as it utilizes sustainable materials, like plants and microbes, which exhibit simplicity, environmental benignity, and low cost (Raheem et al., 2025).

Several metal and metal oxide nanoparticles, such as ZnO_2_, SiO_2_, and Ag_2_O nanoparticles, have been designed for agricultural applications (Yadav et al., 2026a, Yadav et al., 2026b; Amir et al., 2025). Among them, silicon nanoparticles (SiNPs) considered notable due to the quasi-essential role of silicon (Si) for plants. Si, the second most copious element in Earth’s crust, is not essential for plant survival but beneficial as it supports plant growth by mediating adaptation under stress (Chang et al. 2021; Souri et al., 2021; Misra et al., 2023). Si impart numerous beneficial effects to Si hyperaccumulator plants belonging to Cyperaceae and Gramineae family (Kaur and Greger, 2019; Rajput et al., 2021). For instance, rice plant can accumulate nearly 10% Si in its shoot (Kaur and Greger, 2019). The crops such as rice and wheat have active Si uptake mechanisms mediated by Si membrane transporters that support Si absorption at faster rate than the water uptake (Kaur and Greger, 2019). Being crucial for plants, Si fertilizers offer only small-scale benefits to plant growth due to low bioavailability. The Si released from Si fertilizers often polymerizes or forms complexes with metal oxides, reducing uptake by plants (Bhat et al., 2021). However, SiNPs eliminate these challenges and, due to their extraordinary properties, shapes, size, solubility, and high bioavailability, increases the desirability in agriculture (Rajput et al., 2021; Yan et al., 2024). SiNPs are applied to plants as plant growth facilitators, nanopesticides, nanocarriers of biomolecules and chemicals, soil conditioners, and biosensors (Bhat et al., 2021; Rajput et al., 2021; Yan et al., 2024). Silica in soil degrades into non-toxic monosilicic acid, reducing concern related to SiNP-induced environmental toxicity (Miao et al., 2025). SiNPs are taken up by plants in the form of monosilicic acid (Mir et al., 2022), which, after absorption, is subsequently translocated and stored as silicon dioxide (SiO_2_) phytoliths in the cell walls, lumen, and intercellular spaces (Lajmanovich et al. 2018; Verma et al., 2022), providing mechanical support to the plant. They act as a barrier to pathogens, enhance physiological and biochemical processes, particularly photosynthesis and the defense system, and upregulate molecular mechanisms mediating resistance to stresses (Souri et al., 2021; Dorjee and Wangmu, 2023). Si provides resistance against abiotic stresses by alleviating oxidative stress, maintaining nutrient uptake, and lowering water loss via transpiration (Mostofa et al., 2021; Bhardwaj et al., 2023; Dorjee and Wangmu, 2023). For instance, SiNPs positioned at the epidermal cell wall, forming a double subcuticle layer, help in alleviating water loss via transpiration, maximizing water use efficiency under water-deficient conditions (Naidu et al., 2023). Similarly, under salinity stress SiNPs have been reported to alter the gene expression, activating antioxidants enzymes to manage ROS level (Siddiqi et al., 2025). Besides positive interactions, certain studies have documented the toxic effects of SiNPs on plants and ecosystems, attributed to their excess accumulation, as a function of their shape and size (Miao et al., 2025), and/or SiNPs-induced change in the pH of the growth medium (Naidu et al., 2023). However, SiNPs can easily undergo several possible surface modifications, which enable the introduction of changes in their surface chemistry (Naidu et al., 2023), reducing their toxicity (Marzaioli et al., 2014).

Understanding the beneficial and detrimental implications of SiNPs through elucidation of comprehensive molecular mechanisms behind the uptake and translocation, growth enhancement, stress resilience development, and toxic effects in plants is of paramount importance. Thus, the present review aims to provide in-depth information on uptake and translocation of SiNPs in plants and induced changes at the molecular level, particularly SiNP-triggered signaling and activation of defense mechanisms under different stresses to enhance empirical understanding on their role in abiotic stress management and enhancement.

## 2 Bioengineered silicon nanoparticles: properties and biological relevance

**Figure 1:**
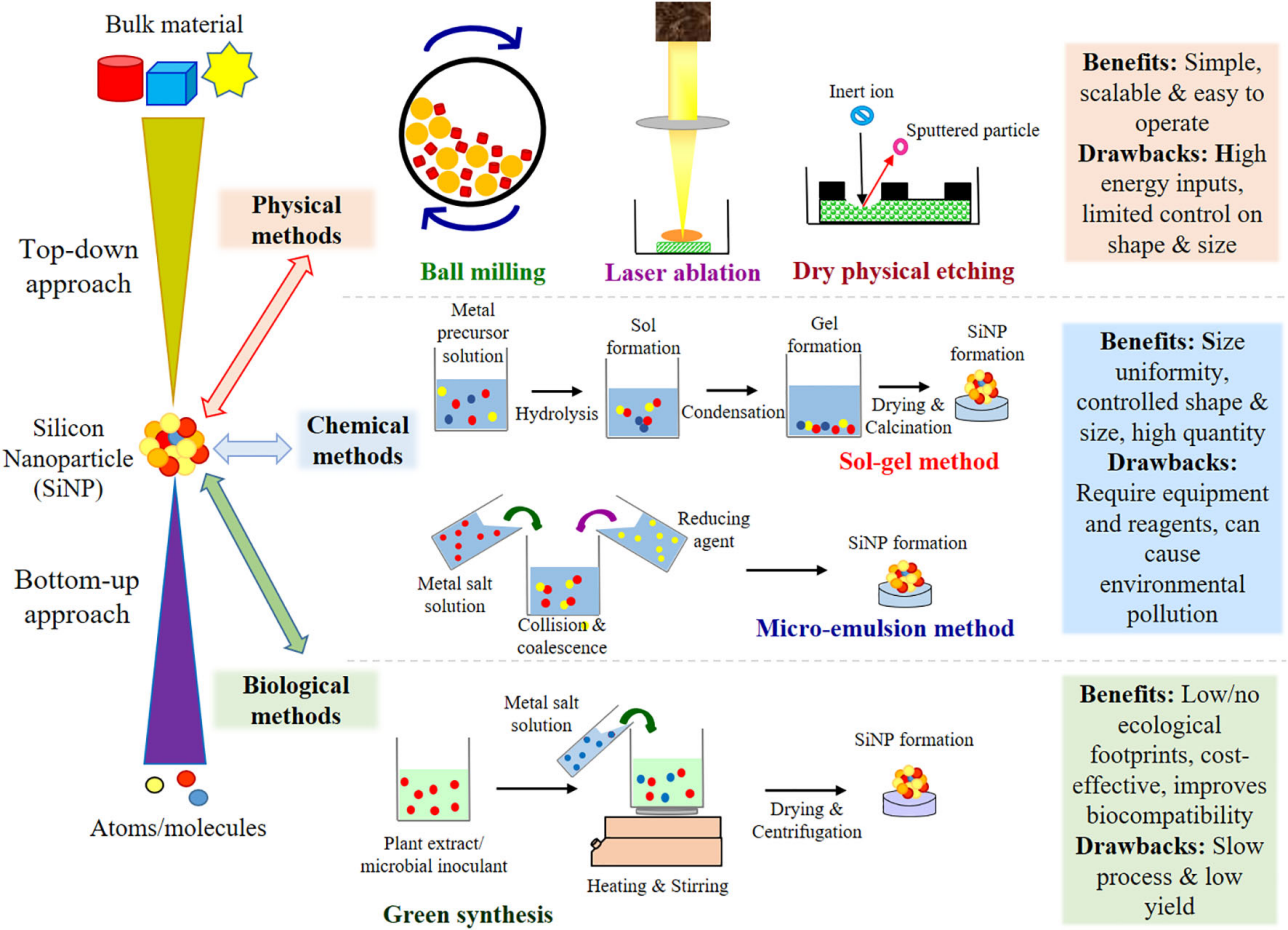
Different approaches for synthesis of silicon nanoparticles and their advantages and disadvantages.

Silicon nanoparticles can be synthesized in varying shapes, sizes, porosity, and crystallinity through a top-down or bottom-up strategy (Naidu et al., 2023). The top-down approach breaks down bulk material into small particles, whereas the bottom-up strategy assembles atoms/molecules into complex nanostructures (Harish et al., 2022; Yan et al., 2024). Based on the driving force and precursors used, the nanoparticles can be synthesized by employing physical, chemical, and biological processes (**Figure 1**). Physical and chemical processes like electrochemical etching, dry etching, ball milling, laser ablation, pyrolysis, sol-gel method, physical vapor deposition (PVD), chemical vapor deposition (CVD), Stober’s approach, magnesiothermic reduction, and reversed microemulsion are ubiquitous methods for the synthesis of nanoparticles (Harish et al., 2022; Yan et al., 2024). These techniques are simple and scalable, facilitating production of SiNPs with desired characteristics (**Table 1**). However, they result in the generation of pollution-causing chemical waste, are expensive, and require high-energy inputs, challenging environmental sustainability (Zamani et al., 2020). Additionally, some of these methods may introduce impurities in synthesized SiNPs, causing structural defects and reducing colloidal stability and functionality (Li et al., 2025). The bioengineering of SiNPs that utilizes plant extracts, microbes (fungi, bacteria, and algae) —natural and/or genetically engineered, industrial/agricultural waste, and protein/peptides-mediated synthesis, can overcome these limitations, resulting in the production of NPs that has low/negligible environmental footprints, are resource efficient, stable, biocompatible, cost-effective, and aid in minimizing agricultural and industrial biowaste (Zamani et al., 2020; Li et al., 2025).

**Table 1:** Biosynthesis of SiNPs using different biological agents.

Biological agent for synthesis	Mechanisms of extraction	Properties/activities of SiNPs	References
Aqueous extract of Euphorbia thymifolia L.	Tetraethyl Orthosilicate (TEOS) was hydrolyzed and condensed through a reaction occurred in the presence of ethanol, HCl (as a catalyst), and plant extract (containing tannins, glycosides, and flavonoids, used as a capping and stabilizing agent). The mixture was continuously stirred, resulting in formation of Si-OH, which further dehydrated and dealcoholized to form SiNPs (Si-O-Si).	The average size of NP synthesized was 17 nm, which reported to improve the percentage of seed germination of Sorghum bicolor at 200 ppm	Periakaruppan et al., 2023
Leaf extract of Punica granatum	Tetraethyl Orthosilicate (TEOS) was mixed with HCl and plant extract (used as a capping and stabilizing agent). The mixture was continuously stirred, and subsequently dried to form SiNPs.	The average size of NPs was reported 12 nm and demonstrated antibacterial activity against two-gram negative pathogenic bacteria	Periakaruppan et al., 2022
Silica-forming peptides	The protein was mixed with the phosphate buffer and subsequently, TEOS was added. TEOS-water biphasic system was stirred continuously, resulting in the formation of SiNPs in the colloidal suspension.	Resulted in synthesis of SiNP with size less than 50 nm	Nguyen et al., 2021
Amyloid fibrils (β-lactoglobulin fibrils)	The Si nanostructure were produced through silicification process using amyloid fibrils, which acted as template for silica deposition. The amyloid fibrils were mixed with the TEOS, resulting in hydrolysis and condensation of the later, leading to fabrication of silicified fibrils	Formed silica core-shell nanofilaments	Cao et al., 2019
Lysine-Leucine peptides arranged with hydrophobic periodicity	Lysine-Leucine peptide was used as a template for synthesis of silica nanostructure. Lysine-Leucine peptide-phosphate buffer saline solution mixed with the silicic acid solution. This lead to the formation of different biosilica nanostructure, depending on the hydrophobic periodicity.	Formed biosilica nanostructure	Baio et al., 2014
Sugarcane bagasse	The silica-rich ash obtained through calcination of sugarcane bagasse was agitated in sodium hydroxide solution to form sodium silicates, which mixed with the HCl to neutralize the pH and incubated for gel formation. The gel was desiccated and dried to obtain the silica powder.	Improved seed germination percentage, plant growth parameters, and protein and chlorophyll content compared to control and inhibited growth of fungal pathogens Fusarium oxysporum and Aspergilus niger	Goswami and Mathur, 2022
Sorghum bicolor leaves	The plant residue was used as silica precursor and silica nanoparticles were fabricated using sequential processes.	Biogenic silica NPs formed were compatible with the colon cells	Athinarayanan et al., 2020
Azadirachta indica leaf extract	The leaf extract mixed with TEOS and stirred overnight, followed by centrifugation. After discarding supernatant, the particles left were dried. The obtained SiNPs were washed, dried, and calcinated at 500 °C.	100–170 nm SiNPs formed showed potential to carry and deliver drug streptomycin, enabling controlled release	Jabeen et al., 2017
Sticky, red, and brown rich husk ash	The ash of rice husk mixed with the HCl to remove metal ions. The obtained ash was then filtered, washed and dried, and further annealed at 700 °C with a ramp rate of 5 °C per min, resulting in synthesis of silica nanopowder.	Three SiNPs (S-SiO2, R-SiO2, B-SiO2) having size 50, 20, and 10 nm, respectively were synthesized using three different forms of rice hull ash	Sankar et al., 2016
Citrus limon L. extract	The silicon precursor solution mixed with Citrus limon extract (used as a reducing agent) and stirred to synthesize water dispersible SiNPs.	Produced SiNPs from two silianes with the average size 20 and 57 nm, respectively	Tiwari et al., 2019
Silico-alumious class F fly ash	Silica was extracted in form of sodium silicate by treating fly ash with sodium hydroxide, which was further converted to silica gel after neutralizing with the HCl by Sol-gel method. The gel was washed, dried, and calcinated.	Synthesized SiNPs with size ranging 20–70 nm, which after calcination and purification resulted in NPs having 90-96% purity	Yadav and Fulekar, 2019
Cynodon dactylon	The turbid solution of plant extract mixed with sodium hydroxide and stirred for 24 h, reaching to pH 12. Then the reaction mixture was titrated with nitric acid to attain pH 8.5-9.0. The solution was centrifuged and supernatant discarded. The remaining content was washed and dried to obtain SiNPs.	Amorphous SiNP formed with average size of particles 60 nm, exhibited antimicrobial property	Babu et al., 2018
Rice husk and Aspergillus parasiticus strain	The biotransformation of rice husk (silica-rich material) by fungus resulted in formation of supernatant which was dried to obtain the SiNPs.	Fungus mediated biotransformation of rice husk-derived amorphous silica into nanoparticles with size ranging from 3 to 400 nm	Zielonka et al., 2018
Saccharomyces cervisiae	The silica precursor was added in yeast solution, followed by centrifugation to separate resultant powder, which was washed with distilled water and centrifuged, followed by series of chemical washing and centrifugation. The resultant solution was dried and treated with hydrogen peroxide to remove floating layers and obtain powder, which again dried to obtain SiNPs.	The SiNPs synthesized determined 5-7% higher oil production/recovery efficiency compared to commercial nanoparticles	Zamani et al., 2020
Californian red worms and rice husk	Bio-digestion of rice husk by worms resulted in humus production, which was dried, pH neutralized, and calcinated at different temperatures. It was subsequently digested to obtain SiNPs.	Worms mediated bio-digestion of rice husk, producing silica, which on calcination produced SiNPs of size 55–250 nm	Estevez et al., 2009

The biological extracts or cells contain enzymes and several biologically active molecules such as hormones, vitamins, proteins, peptides, organic acids, alkaloids, and polyphenols, biosurfactants, which act as reducing or stabilizing agents, facilitating the synthesis of NPs (Jalil et al., 2019; Goswami et al., 2022; Harish et al., 2022; Yadav et al., 2025). Certain reductases present in the plant extract or cell can change silicate precursor into amorphous silica nuclei. The functional groups present on the surface of such biomolecules act as a capping agent, which, on interaction with the silica nuclei, encase the NPs, improving their stability, function, and biocompatibility (Li et al., 2025). The biosurfactant—rhamnolipid-modified SiNPs were reported to inhibit the phytopathogen *Magnaporthe oryzae*, the causal agent of blast disease in rice. The functionalized SiNP was documented to reduce disease incidence by 10.80%, the relative growth of the pathogen by 97.05%, while improving the shoot dry biomass by 13.33% compared to the control (Li et al., 2024). The biosynthesis of SiNPs operates at mild conditions, requires less cost, eliminates the risk of toxic waste generation, and reduces energy inputs, making the approach sustainable and resource-efficient (Zamani et al., 2020; Harish et al., 2022; Li et al., 2025). Bansal et al. (2005) biosynthesized SiO_2_ NPs from anionic complex SiF_6_^2-^ by using the fungus *Fusarium oxysporum*. Similarly, Show et al. (2015) biosynthesized SiNP from magnesium trisilicate and tetraethyl orthosilicate in a cost-effective manner using BKH1 bacteria, avoiding the complex multi-step process of SiNP formation. Natesan et al. (2021) investigated the toxic effect of SiNPs biosynthesized using *Trichoderma atroviride*, *Pseudomonas fluorescens*, and *Streptomyces griseus* on Zebrafish. In the study, no harmful effects were reported in the model organisms exposed to 0.5, 3, and 30 µg concentrations of SiNP. The agricultural and industrial biomass waste, like corn-cob, corn-stalk, rice and wheat husk, coconut hulls, fly ash, glass waste, palm kernel shells, and sugarcane bagasse, are silica-rich materials that contain high content of silica (Akhayere et al., 2022; Malpani and Goyal, 2023; Li et al., 2025). These low-cost materials are utilized to produce high-grade silica precursor through approaches such as the sol-gel method, which generates SiNPs (Dorairaj et al., 2022; Li et al., 2025). Adebisi et al. (2021) synthesized agro-waste (cassava periderm, corn-cob, and corn-stalk) based SiNPs by utilizing a modified sol-gel method along with metallothermic reduction. Analogously, Dorairaj et al. (2022) synthesized mesoporous SiNPs from UKMRC-8 rice husk utilizing the sol-gel method. This method helps in minimizing the problem of industrial and agricultural waste management, utilizes cheap and non-toxic sources for the production of silica precursor, implements a circular economy, and eliminates the burning of agro-waste, thereby reducing carbon footprints. Moreover, the synthesized SiNP retains traces of bioactive elements of biomass waste, which improves their agricultural efficiency (Akhayere et al., 2022; Dorairaj et al., 2022; Li et al., 2025).

The peptides/proteins, such as bovine serum albumin (BSA), silaffin, and silicon-forming peptides (SFP) occurring in living organisms like diatoms, brown algae, and sponges, mediate the formation of silica structures under normal physiological temperature and pH conditions by the biosilicification process (Wang et al., 2014; Abdelhamid et al., 2024). These biomolecules, as well as their bioengineered variants like R5 peptide, can serve as a biotemplate and mediate biomimetic synthesis of SiNPs in *in-vitro* conditions, resulting in SiNP production for use in drug delivery, photonics, agriculture, etc (Jackson et al., 2015; Nguyen et al., 2021; Reichinger et al., 2022; Abdelhamid et al., 2024). Jackson et al. (2015) used BSA for the green synthesis of SiNPs. The SiNPs synthesized had a size ranging between 250 and 380 nm and displayed reversible and ionically binding ability with other proteins. The use of lysine-leucine peptides with varyingly arranged hydrophobic periodicity resulted in the synthesis of SiNPs with diverse nanostructures, including rod-shaped structures, wire-like structures, and nanospheres (Baio et al., 2014). Wong Po Foo et al. (2006) synthesized a silicon nanocomposite using a chimeric protein consisting of the R5 peptide and spider silk protein. Bioengineering of SiNPs is a promising approach for fabricating materials that can enable sustainable agricultural activities, reducing intensive reliance on agrochemicals, and supporting food production under increasing pressure from biotic/abiotic stresses (Rastogi ett al. 2019).

## 3 Silicon nanoparticles-mediated plant growth and stress alleviation

### 3.1 Uptake, translocation, and accumulation of SiNPs in plants

**Figure 2:**
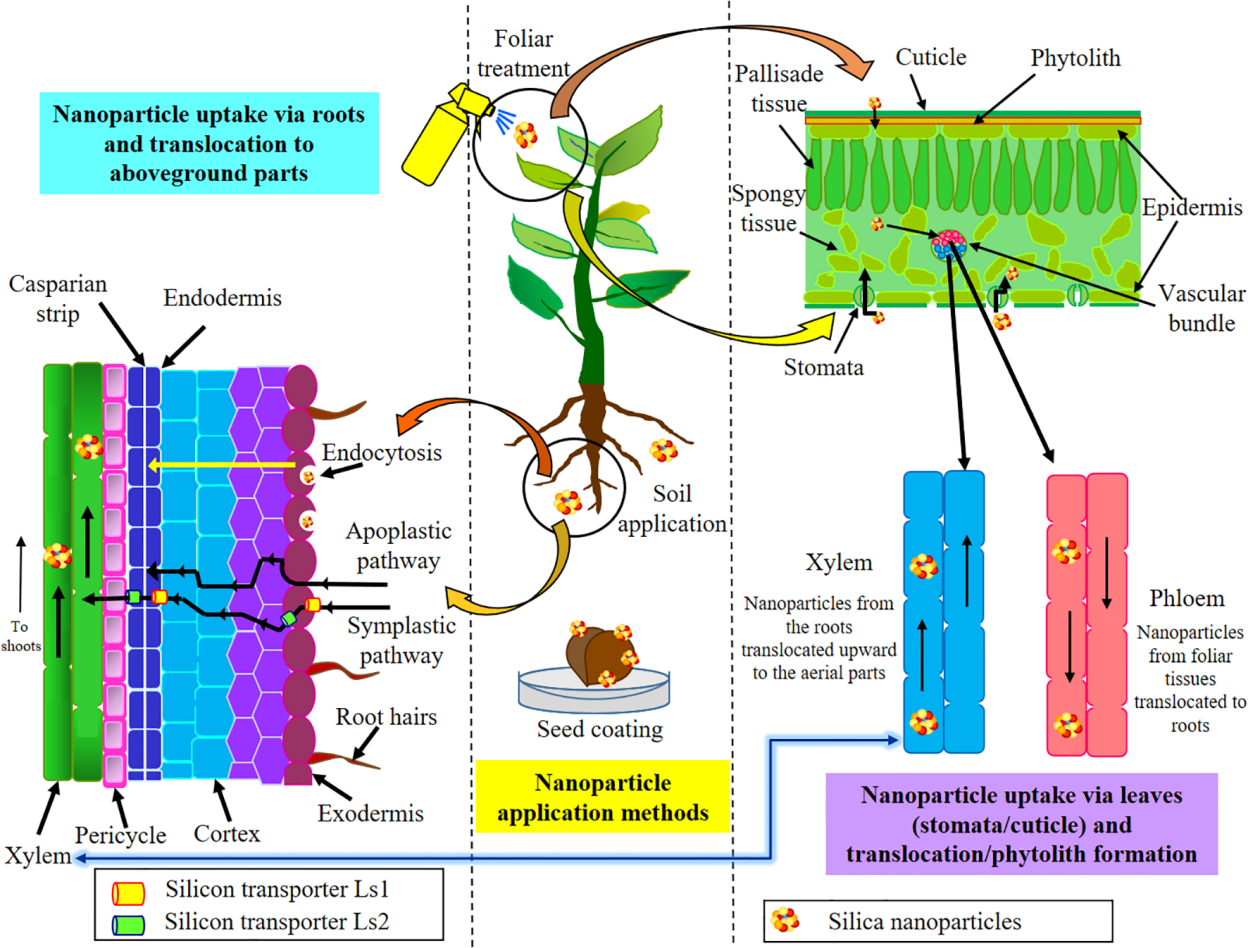
Various methods of silicon nanoparticles application, uptake and translocation mechanism in plants.

Bulk Si and SiNPs both induce a positive effect on plants under normal and stressed conditions. However, SiNPs have been evidently reported to have more benefits than conventional bulk counterparts owing to their smaller size, high surface/volume area, and more bioavailability, which enables them to be swiftly absorbed by the plants and translocate to distal parts. These NPs enter plants either passively or actively through root cells, cuticle/stomatal openings, and/or the base of trichomes, and translocated/distributed to different parts, facilitated by plasma membrane localized transporters viz., Low Silicon1 (*Lsi1*), Low Silicon2 (*Lsi2*), and Low Silicon 6 (*Lsi6*) (Goswami et al., 2022; Yan et al., 2024; Maurya et al., 2025) (**Figure 2**). The plants that are poor or non-accumulators of Si and lack specific Si transporters are also get benefited by SiNPs, as their small size allows direct movement inside the plant cell via stomata and the cell wall (Bhat et al., 2021).

SiNPs are administered in plants via nutrient growth solution/root zone application, foliar spray, and seed priming (Maurya et al., 2025). SiNPs sprayed on the above-ground parts enter the plant via the cuticle/stomata. SiNP can also get absorbed via root cells when supplied through soil/nutrient solution. The uptake of SiNPs by plants depends on multiple factors, such as the morphology and charge of SiNPs, mode of application, concentration of NPs, physicochemical properties of growth media, pore size of the cell wall, plant species, and plant growth stage (Azim et al., 2023; Goswami et al., 2022; Yan et al., 2024). In *Arabidopsis thaliana*, up to 200 nm size SiNPs were absorbed by the roots (Slomberg and Schoenfisch, 2012). Studies have shown that soil application is more effective for the uptake of SiNPs than foliar spray due to the higher absorption rate, owing to the large pore size and presence of Si transporters (Bhat et al., 2021). Another mode of SiNPs application to plants is seed priming (nano-priming). This approach has an advantage over all other methods, as it imbues seeds with hydration before germination and upregulates the early physiological state of seeds (Bhat et al., 2021). This improves the seed germination rate and seedling growth (Hatami et al., 2021) as reported in *Helianthus annuus* (Janmohammadi and Sabaghnia, 2015).

SiNPs in soil are dissolved as monosilicic acid, which plants take up via symplastic and apoplastic pathways (involving plasmodesmata and cytoplasmic bridges) (Bhat et al., 2021). The SiNPs, which remain in the insoluble form, are taken up by endocytosis, a transmembrane pathway formed by the folding of the cell membrane (Azim et al., 2023). The root exudates released by plants contain several metabolites that bind with SiNPs, forming complexes, which are subsequently attached to membrane transporters/carrier proteins and eventually absorbed via ion channels/aquaporins (Bhat et al., 2021). The root-to-shoot and shoot-to-root transportation occur via xylem and phloem, respectively. The small-sized SiNPs (20nm) enter plant roots via apoplastic and symplastic pathways (penetrate the cell wall and then move to the endodermis and intracellular spaces) and eventually translocate to aerial parts via xylem conducting tissues (Sun et al., 2014). Ls1 transporter, which is the nodulin-26 major intrinsic protein (NIP), is localized at the distal side of exodermis and endodermis cells of roots and facilitates the passive influx of silicon from the apoplast to the root cell. The active efflux of Si from the root cell to the xylem vessel, known as xylem loading, is mediated by Ls2, which is localized on the proximal side of the exodermis and endodermis cells of the roots (Rajput et al., 2021). Monosilicic acid from the xylem is transported to the shoot by the transpirational pull, and subsequently unloaded to the leaf epidermal cells (Mukarram et al., 2024). In rice, Ls6 has been reported to facilitate the unloading of Si from xylem and its deposition in shoots (Yamaji et al., 2008). In the aerial part of the plant, an increase in the content of monosilicic acid mediates polymerization, which changes to amorphous silica gel (Mukarram et al., 2024).

### 3.2 SiNPs-mediated plant growth and development under stress

Silicon nanoparticles have a multifunctional role in agriculture. These NPs directly or indirectly induce positive effects on plants. SiNPs directly stimulate plant growth by enhancing nutrient uptake, mitigating biotic stresses, and imparting tolerance to abiotic stresses. SiNPs also facilitate the precise and controlled delivery of agrochemicals, which indirectly improves plant growth, enhances efficiency, and reduces the input of fertilizers and pesticides (Sarkar et al., 2022). The use of biosynthesized SiNPs holds exciting prospects over conventional physical and chemical methods-derived SiNPs due to the presence of bioactive trace elements, which eventually improve their functionality (Li et al., 2025).

SiNPs, after absorption and translocation in the plant, induce cellular and physiological changes, such as improving nutrient absorption, enhancing phytohormone synthesis, and increasing antioxidant content, which ultimately render oxidative stress and modulate signaling processes, augmenting stress tolerance and plant yield (Miao et al., 2025). Small-sized SiNPs can penetrate the seed coat, promoting seed germination, plant growth, and overall development. SiNPs after accumulation in the epidermal cell wall causes cell wall modification by forming a double layer through crosslinking with hemicellulose. The layer acts as a physicochemical barrier, providing strength to the plant, preventing water loss via transpiration, thereby improving root hydraulic conductivity and preventing desiccation under water-deficient conditions, and inhibiting invasion of pathogens and entry of toxic elements (Goswami et al., 2022; Ahmed et al., 2023; Miao et al., 2025). Thus, SiNPs act as a fertilizer, reduces biotic stress, and ameliorate the impact of abiotic stimuli. For instance, application of 8 gL^-1^ SiNP to tomato seeds was observed to improve the seed germination percentage (22.16%) and index (22.15%), mean seed germination time (3.98%), seedling vigor index (507.82%), and seedling fresh and dry weight (116.58% and 117.46%), compared to the control (Siddiqui and Al-Whaibi, 2014). Similarly, treatment of maize seeds with SiNPs grown in hydroponic and pot conditions was found to have improved germination percentage, increased silicon accumulation, and a higher content of other nutrients in the seeds compared to their bulk counterparts (Suriyaprabha et al., 2012). Treatment of soil with 200 mg/L of SiNP significantly improved germination percentage (28.7%), vigor index (46.7%), germination index (68.8%), and germination speed (70.3%) of cucumber compared to the control (Alsaeedi et al., 2019). Similarly, soaking sunflower seeds in different concentrations of SiNPs (0.2, 0.4, 0.6, 0.8, 1.0, 1.2 mM) for 8 hours improved the seed germination percentage, mean germination time, seedling length, and seedling vigor index (Janmohammadi and Sabaghnia, 2015).

**Figure 3:**
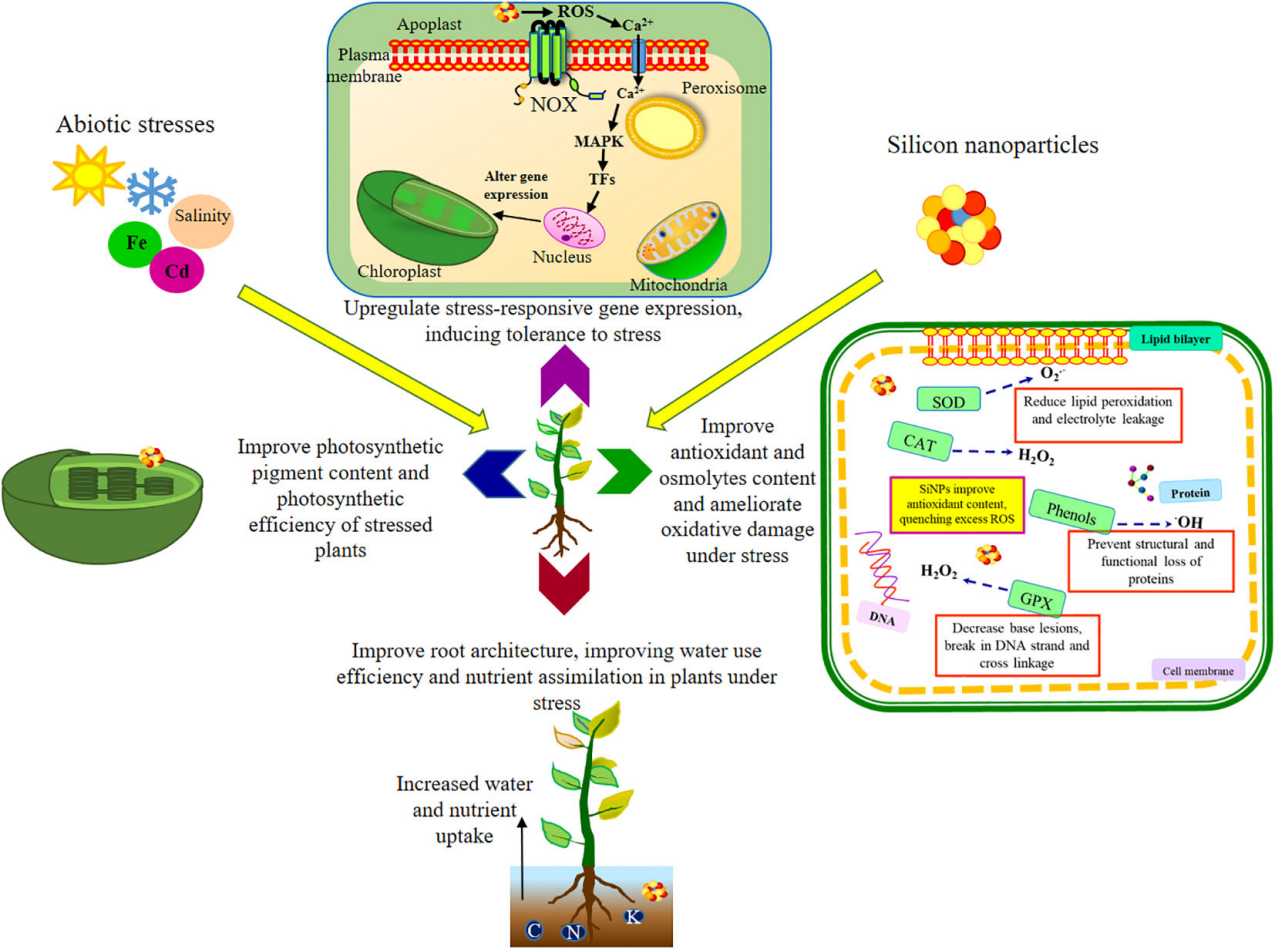
Mechanism of silicon nanoparticles mediated mitigation of adverse effects of abiotic stress and improved plant growth.

SiNPs improve photosynthesis by promoting absorption, transmission, and transformation of light energy at the photosystem (PS) II reaction centre, increasing the rate of electron transport at PSII, and synthesis of photosynthetic pigments and related enzymes (Mukarram et al., 2024). It upregulates the expression of genes encoding proteins involved in the photosynthetic process. Moreover, SiNPs stimulate the population of plant growth-promoting microbes in soil, thereby improving plant growth and nutrient availability (Mukarram et al., 2024). SiNPs improve antioxidant content in plants, quenching excess ROS and thereby curtailing oxidative damage (**Figure 3**). The effect of SiNPs application on the growth and yield of lemongrass was investigated by Mukarram et al. (2021), who observed an upregulation in photosynthetic activity, antioxidant metabolism, and the activity of the enzymes geraniol dehydrogenase and nitrate reductase, crucial for essential oil production and nitrogen metabolism, respectively, at 150mgL^-1^ concentration.

SiNPs can support plant growth and manage stress by efficiently delivering bioactive molecules and/or agrochemicals such as pesticides, fertilizers, phytohormones, siRNA, and other genetic materials through entrapment in the cavities or pores of the mesoporous structure (Sarkar et al., 2022; Cai et al., 2024). The delivery of siRNA in plants can facilitate long-term silencing of genes, improving plant health and growth (Cai et al., 2024). Yi et al. (2015) fabricated ultrafine mesoporous silicon nanoparticles to deliver salicylic acid (SA) in plants, which exhibited redox-responsive release of SA under *in vitro* conditions.

## 4 Interaction of soil-silicon nanoparticle-plant

**Figure 4:**
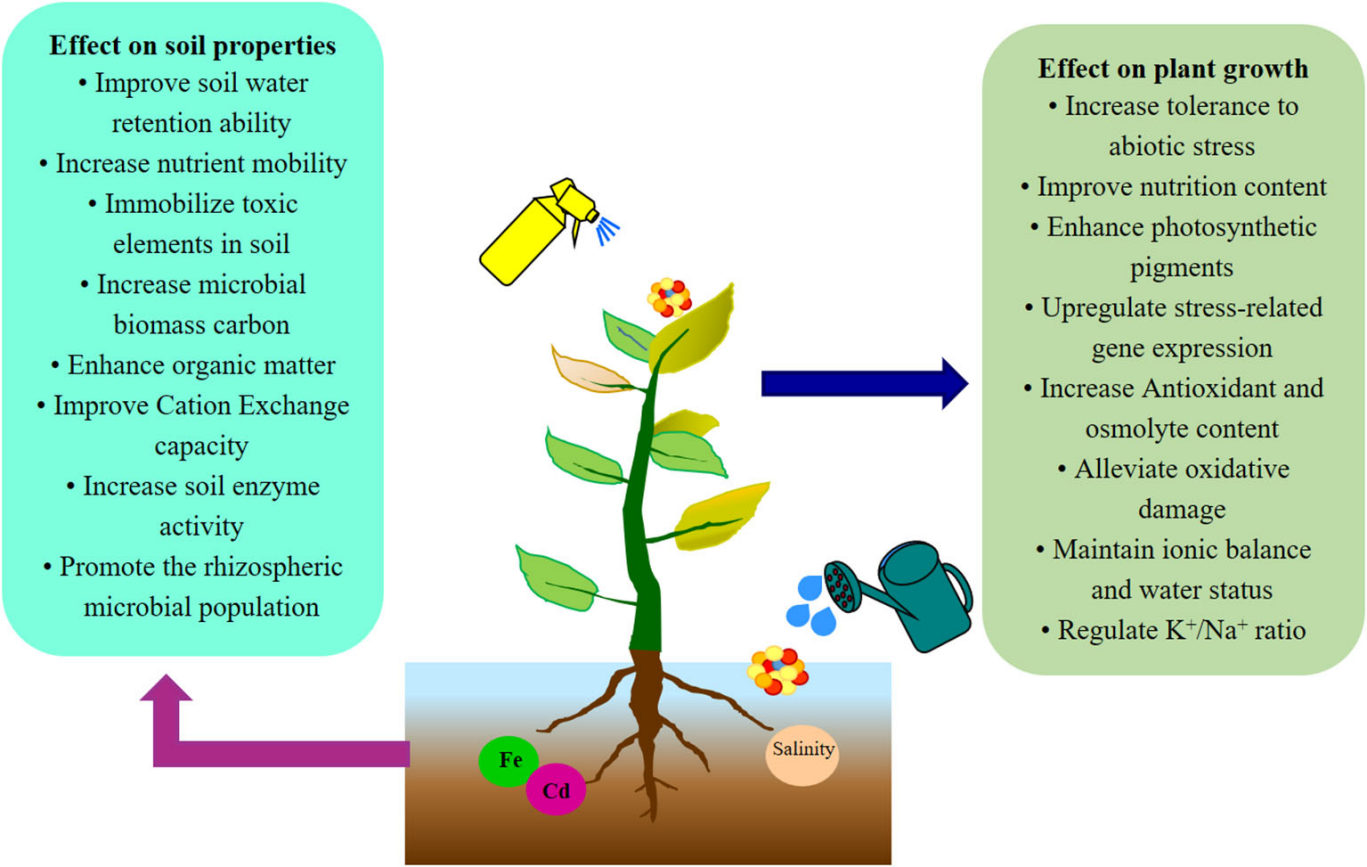
Interaction of biosynthesized silicon nanoparticles with soil and plant under stress and effect on soil properties and plant growth.

SiNP applied to the soil or plants interacts intricately with the soil particles, microorganisms, and plants, influencing soil characteristics and plant development (**Figure 4**). The physicochemical and biological properties of soil such as soil moisture, pH, cation exchange capacity (CEC), and microbial population determines the soil fertility. The presence of contaminants, toxic ions, and/or environmental stresses can severely alter soil characteristics, affecting food production. Therefore, sustainable and long-term mitigation approach is needed. Treatment of soil with SiNPs has demonstrated effective role in alleviation of stresses like salinity, heavy metals, and drought, and improvement in plant growth by stimulating microbial community structure, remediating contaminated soil, and promoting nutrient availability. For instance, the application of different functionalized forms of nano-silica on metal (Cd, As, Pb, and Cu) contaminated soil reduced leachability and bioavailability of mobile metals by transforming them into immobile and stable fractions, facilitating bioremediation of soil (Cao et al., 2020; Wang et al., 2014; Lian et al., 2021). Moreover, after bioremediation of metal contaminated soil by functionalized nano-silica, increase in microbial biomass carbon, organic matter, and CEC in soil and decrease in soil bulk density have been reported (Lian et al., 2021). SiNP was observed to improve water-use efficiency in soil by managing field capacity, available water content, wilting point, and saturation water content (Surya et al., 2025). The improvement in water retention capacity of soil was found by El-Aziz et al. (2025) on addition of nano-silica hydrogel, which increased soil water retention capacity, crop productivity, and crop water productivity, under reduced water conditions. The structure and activities of rhizospheric microorganisms in soil such as phosphate-solubilizing bacteria (PSB) and nitrogen-fixing bacteria, can also be influenced by the presence of SiNPs (Tian et al., 2020; Panigrahi and Rout, 2025). The application of SiNP in soil has been reported to improve the soil nutrient status, soil enzyme activities, as well as bacterial diversity in wheat field (Zhu et al., 2024). Application of 8 nm size SiNP at 50 mg/kg to paddy soil, improved plant tillers and grain yield significantly over control and sodium silicate by increasing N content in plant roots and shoots, optimizing root exudates, improving rhizospheric bacterial population, increasing cytokinin/auxin ratio, reducing gibberellic acid content, and upregulating gene expression related to tillers like *OsMOC*, *OsFON*, and *OsTB* (Yue et al., 2023). Even the foliar application of tomato plant with 100 mgL^-1^ SiNP, subjected to low temperature stress, displayed enhanced tolerance toward cold as a result of improved root architecture, photosynthetic activity, antioxidant capacity, nutrient metabolism, absorption, and use, and improved rhizospheric microbial community structure, facilitating soil nutrient release (Shi et al., 2025). The work undertaken by Tian et al. (2020) demonstrated that the foliar application of SiNP on Pakchoi plant grown over contaminated mine soil, significantly increased soil metabolite profile including sugars, phenolic compounds, small organic acids and fatty acids (while decreasing amino acid) through release of root exudates, facilitating increase in bacterial (*Paenibacillus*, *Pseudonocardia*, *Skermanella*, *Ferruginibacter*) and fungal (*Chaetomium* and *Ilyonectria*) genera in rhizosphere that were related to carbon and nitrogen cycles. SiNPs offer numerous advantages to soil and plants by supporting growth of beneficial microbial population, improving soil physicochemical properties, increasing nutrient availability, immobilizing toxic elements, and altering gene expression and phytohormone/antioxidants level under both normal and stressed conditions. Promoting SiNPs use in agriculture could possibly guarantee food security amid climate change crisis by ameliorating abiotic stresses, improving soil characteristics and promoting plant-microbe partnership.

## 5 Alleviation of abiotic stress in plants by biosynthesized SiNPs

Silicon nanoparticles induce tolerance in plants against various abiotic stresses, occurring either individually or simultaneously (Roy et al., 2025a). SiNPs mitigate adverse effects by triggering appropriate responses at cellular and molecular levels.

### 5.1 Role in alleviation of heavy metal-induced stress

Heavy metals, whether essential or non-essential, are potentially toxic elements that may induce phytotoxicity upon accumulation above their threshold concentrations (Sachdev et al., 2025). Suppressed plant growth, disrupted photosynthesis, and the generation of excess ROS, leading to oxidative stress are characteristic effects of heavy metal-induced toxicity (Li et al., 2025). SiNPs can prevent these damages by restricting metal uptake and translocation in plants through the formation of complexes in the soil (Mukarram et al., 2024). Si possesses excellent ability to form chelates with the soluble elements (El-Saadony et al., 2021). SiNPs can also ameliorate damage to the photosynthetic process by improving the content of photosynthetic pigments, i.e., chlorophyll and carotenoids (Li et al., 2025). It reduces oxidative stress by activating antioxidant machinery and upregulating gene expression associated with plant physiological and anatomical activities (Mukarram et al., 2024). For instance, the treatment of bamboo species with 500µM SiNP improved plant growth, photosynthesis, and antioxidant activity, and alleviated hydrogen peroxide content (H_2_O_2_) and lipid peroxidation under Pb stress (Emamverdian et al., 2020). The biosynthesized SiNPs applied through foliar spray on *Phaseolus vulgaris* L., grown on the saline soil contaminated with heavy metals, improved the growth of the plant, photosynthetic efficiency, antioxidant content, nutrient content, Na^+^ and K^+^ ratio, membrane stability, free proline level, total soluble sugar, stomatal conductance, relative water content, and transpiration rate. The application of 05 mmol of Biosynthesized SiNP significantly mitigated saline soil and heavy metals-induced oxidative damage in *Phaseolus vulgaris* L. and reduced Cadmium (Cd), Lead (Pb), and Nickel (Ni) accumulation in leaves and pods (El-Saadony et al., 2021). Treatment of rapeseed with 250 mg kg^-1^ biosynthesized SiNPs subjected to Cd stress, displayed significant improvement in photosynthetic efficiency, fresh and dry weight, Si uptake, and activity of antioxidants with reduction in Cd accumulation, alleviating metal-induced oxidative damage and toxicity (Ahmed et al., 2023). Analogously, SiNPs can modulate the expression of heavy metal transporter genes, transcription factors (TFs), and genes related to antioxidant enzymes (Huang et al., 2024). For instance, *Brassica napus* subjected to Chromium (Cr) stress demonstrated an increase in the expression of genes associated with antioxidant enzymes Superoxide dismutase (SOD), Catalase (CAT), Peroxidase (POD), and Ascorbate Peroxidase (APX) and the stress-responsive TFs *WRKY*, *CPX*, and *MYC*, and reduced expression of metal transporter genes (sugar transporters (*ST1* and *ST8*), ATP-binding cassette (*ABCG37*), HM ATPase (*HMA*), and metallothionein (MT), reducing Cr uptake and phytotoxicity (Huang et al., 2024). The application of SiNPs in combination with FeNPs in rice alleviated Pb-induced toxicity by significantly improving the content of antioxidants (SOD, POD, Glutathione (GSH), and CAT) (Ghouri et al., 2024). It was noticed that both NPs induced a synergistic effect, leading to an alteration in gene expression related to metal transporters and antioxidants. In rice plants, an increase in the Si content was found to promote co-precipitation of Cd in the cell wall of the plant, thereby reducing the concentration of Cd in the apoplastic and symplastic pathways (Ma et al., 2017). David et al. (2024) investigated the role of green-synthesized SiNPs in mitigation of arsenic-induced stress in maize plants. In the study, an increase in the production of phytochelatin (PCs) in plant root exudates was reported, which mediated the formation of the SiNP-As-PCs complex, stabilizing Arsenic (As) in maize roots. SiNPs were found to reduce the oxidative damage by activating ROS scavenging– antioxidant defense system, stimulating deep and extensive root structure, and improving root hydraulic conductance, eventually resulting in increased plant growth and development. Similarly, green-synthesized SiNPs alleviated Cr stress-induced phytotoxicity in tomato plants by improving root architecture, photosynthetic rate, leaf gaseous exchange, and antioxidant activities through upregulation of gene expression (Altaf et al., 2024). Another study undertaken by Malik et al. (2025) reported attenuation in Cd uptake by melon plants and reduced toxicity when supplied with 75 mg L^-1^ biosynthesized SiNP. The improved content of chlorophyll and carotenoid, antioxidant activity, macronutrient uptake, and upregulated expression of stress-related genes significantly stimulated plant growth parameters.

### 5.2 Salinity stress alleviation and plant growth by SiNPs

Salinity is one of the most severe abiotic stresses that abridge plant growth and productivity. Soil salinity induces osmotic stress, ion toxicity, and ROS-induced oxidative damage in plants, disrupting membrane integration and increasing electrolyte leakage. It also hampers the nutritional balance and photosynthetic process by reducing the availability of CO_2_ due to increased stomatal closure, inducing toxic effects on photosynthetic apparatus/rate, as well as decreasing the leaf surface area (Haghighi and Pessarakli, 2013). Salinity prompts the accumulation of salt in the apoplast, causing cell dehydration and inhibiting the activity of enzymes involved in plant metabolism (Haghighi and Pessarakli, 2013). NPs can alleviate the stress in plants by modifying mineral uptake and reducing ROS concentration (Iqbal et al., 2020). For instance, Roy et al. (2025b) reported improvement in the growth of lettuce growing under salinity-stressed conditions on exogenous application of ZnO NPs by improving K^+^ content and K^+^/Na^+^ ratio and alleviating oxidative stress. Si can induce an ameliorative effect on plants under salt stress. Si after absorption by plants undergo lignification, suberization, and silicification in cell wall, and act as a barrier, restricting Na^+^ uptake and translocation to the aerial part, thereby alleviating their concentration in cellular compartment. Si also mitigate salinity-induced oxidative stress by increasing antioxidants production and stimulating stress tolerance pathway by upregulating gene expression (Manimaran et al., 2025). Zhu et al. (2019), reported silicon mediated upregulation of gene expression like MYB44-like transcription factor (TF), AP2 domain TF RAP2, and auxin-induced protein, responsible for photosynthesis, oxidative stress alleviation, and auxin signaling, in cucumber imparting tolerance to salinity stress. Similar to Si, SiNPs after uptake, induces tolerance to salinity stress by preventing accumulation of Na^+^ and Cl^–^ via regulation of ion transporters, mediating sequestration and vacuolar compartmentalization of Na^+^, increasing K^+^ retention, maintaining K^+^/Na^+^ ratio, improving structural enforcement, promoting water uptake and/or by increasing production of antioxidants via elicitation of signaling pathways, upregulating TFs and responsible genes (Sajida et al., 2025; Shoukat et al., 2025). SiNPs have been reported to induce the production of osmolytes in plants under salt stress, maintaining osmotic homeostasis and turgor pressure, and maintaining ionic balance by restricting Na^+^ uptake and promoting K^+^ influx through modulation of membrane transporters (Yin et al., 2016). For instance, the application of SiNP to the lentil seedling exposed to salt (NaCl) stress reduced oxidative damage by increasing the content of non-enzymatic antioxidants, improved germination, and reduced Na^+^ uptake, thereby maintaining the Na^+^/K^+^ ratio (Sarkar et al., 2022). Pinedo-Guerrero et al. (2020) reported that the application of SiNPs of size ranging 10-20nm enhanced the production of glutathione and β-carotene content in tomato plants exposed to salt stress. In many plants, such as wheat and maize, the Si-mediated tolerance to salt stress has been observed (Ahmad et al., 1992; Parveen and Ashraf, 2010), which may be imparted due to improved photosynthetic activity, antioxidant content, water status, and changes in leaf ultra-structure, and reduced membrane permeability and specific ion availability. Improvement in water use efficiency, stomatal conductance, and photosynthetic rates was observed in cherry tomato plants on application of both Si and SiNPs, triggering tolerance to salinity stress (Haghighi and Pessarakli, 2013). Similarly, in maize, seed priming with SiNP reduced the effect of alkaline stress by improving leaf relative water content, increasing photosynthetic pigments, and enhancing potassium ions, total free amino acids, and soluble sugar and protein content (Alsamadany et al., 2024). The double layer formed by silica deposition on the epidermal cell wall also prevents water loss and helps to maintain the osmotic pressure under salt stress. The cotton seedlings (malvaceae family) exposed to the salt and low-temperature stress simultaneously showed significant reduction in the growth parameters (Liang et al., 2023). The foliar application of SiNPs upto 100 mg L^-1^ reduced the damage by improving the photosynthetic efficiency, electron transport activity of photosystem II, chlorophyll content, stomatal conductance, stomatal size and density, transpiration rate, and activities of Rubisco enzyme and phosphoenolpyruvate carboxylase in addition to decrease in abscisic acid (ABA) and malondialdehyde (MDA) content (Liang et al., 2023). The SiNPs concentration beyond 100 mg L^-1^ decreased the seedling growth. The stress-induced osmotic stress, increased ABA content (responsible for regulating stomatal opening and photosynthetic activity), and oxidative damage was probably alleviated by improving K^+^/Na^+^ ratio, restricting the expression of genes involved ABA synthesis pathway and production of ROS-scavenging enzymatic and non-enzymatic antioxidants by SiNPs (Liang et al., 2023, Liang et al., 2024). Application SiNP also stimulate the plant microbiome, which influence the plant even under stressed conditions. For instance, soybean plants exposed to salinity stress, was treated with SiNP to assess the role of nanoparticles on the microbial community in rhizosphere and endosphere (roots and leaf) (Wang et al., 2024a). The experiment revealed the strong and positive response of SiNP foliar application on bacterial community enrichment in endosphere (root–13 microbial taxa and leaf–24 microbial taxa), however, in rhizosphere (2 microbial clades) low community enrichment was observed. The beneficial bacterial genera including *Pseudomonas*, *Bacillus*, and *Variovorax*, enriched by the SiNPs supported plant growth and tolerance to the salinity stress. Change in rhizospheric community structure, increase in microbial population by SiNP and improved plant growth signifies the synergistic role of microbes and SiNP in management of salinity stress.

### 5.3 SiNPs improves plant growth and tolerance under drought stress

Drought stress reduces water availability to plants, affecting water potential and turgor pressure, impairing physiological and metabolic activities. Drought stress is characterized by increased stomatal closure, reduced gaseous exchange and nutrient content, enhanced photoreduction in the chloroplast, and elevated photorespiration (Ansari and Lin, 2010; Sachdev et al., 2021; Raza et al., 2023). These alterations trigger overproduction of ROS, eventually hindering plant growth and yield. Si and SiNPs have immense potential to rescue plants like wheat, tomato, and others from drought-induced physiological assault by improving relative water content, nutrient assimilation, photosynthetic content, and ameliorating the impact of oxidative damages through elevated antioxidant activities (Raza et al., 2023). In particular, wheat crop suffering from drought stress demonstrated significant improvement on application of 60 ppm biosynthesized SiNPs. It reduces H_2_O_2_ and MDA content, improved antioxidant activity, relative water content, proline, and chlorophyll levels, and upregulated expression of stress-responsive genes, viz., DREB2, MYB33, MYB3R, WRKY19, and SnRK2.4 (Boora et al., 2023). The wheat seed priming with 900 mgL^-1^ SiNP grown under drought stress improved plant growth parameters, biomass, yield, and chlorophyll and carotenoid content, and reduced oxidative stress and electrolyte leakage compared to the control by increasing the content of antioxidants (SOD and POD) (Raza et al., 2023). Seed priming with SiNPs at a concentration of 250 mgL^-1^ reduced the impact of drought stress and imparted tolerance to *Elymus sibiricus* by attenuating oxidative damage and improving enzymatic antioxidants, osmolytes, and chlorophyll content (Khan et al., 2024). Eggplant grown at a drought level of 60% crop evapotranspiration significantly suffered growth and yield loss, which was eventually alleviated on application of 300 mgL^-1^ biosynthesized SiNPs due to improved antioxidant levels, photosynthetic activity, relative water content, and ionic homeostasis. SiNP reduced H_2_O_2_ accumulation and lipid peroxidation by increasing the activities of POD, SOD, and CAT (Younes et al., 2024). In a recent study, application of SiNPs improved maize productivity and water-use efficiency under drought through enhanced photosynthetic performance and canopy light utilization (Liang et al., 2026).

### 5.4 SiNPs mediated alleviation of heat stress effect on plants

High temperature induces heat stress in plants and has become a common stressor in the present era due to the gradual increase in global warming. Heat stress affect plants at the metabolic, morphological, and cellular levels, causing structural dysfunction of the cell organelles, particularly the photosynthetic apparatus (Sachdev et al., 2021). Heat stress exacerbates the production of ROS in plants, compromising membrane stability and enzymatic activities (Sachdev et al., 2021). Moreover, heat stress increases water loss due to the excess transpiration, affecting plants’ water status. The application of SiNPs has shown a promising effect in alleviating heat stress-induced damage and growth restriction in plants by upregulating the expression of stress-responsive genes, modulating the antioxidant defense system, and improving the ultrastructure and function of cell organelles, relative water content, and nutrient homeostasis. For instance, the treatment of wheat seedlings with SiNPs demonstrated amelioration of heat stress-induced injuries. SiNPs restored the ultrastructure of the chloroplast and nucleus, and improved the rate of photosynthesis by enhancing the photosynthetic efficiency of PS II, photosynthetic pigment content, and the performance index (Younis et al., 2020).

## 6 Molecular mechanism of bioengineered SiNPs mediated plant growth under abiotic stress

Plants subjected to stresses show limited growth due to the disruption of normal physiological and metabolic activities. The perception of abiotic stresses increases the generation of ROS in plants, suppressing the innate defense system. ROS with affinity toward target biomolecules trigger a series of chain reactions, damaging lipid membranes, crucial proteins, enzymes, carbohydrates, and nucleic acids (Sachdev et al., 2021, Sachdev et al., 2025). Biosynthesized SiNPs have been observed to mitigate the effects of stresses in plants by alleviating the concentration of ROS, thereby improving their growth and development. For instance, the treatment of tomato seeds with SiNPs under salt stress improved seed germination percentage and fresh weight, reduced oxidative stress by enhancing antioxidant activities, upregulated genes responsible for the synthesis of Gibberellin (GA) and catabolism of Abscisic acid (ABA), and downregulated genes deactivating GA and the synthesis of ABA (Wang et al., 2024b). SiNPs also improved the water-use efficiency of plants by developing a deep and branched root structure, reducing the transpiration rate, promoting the photosynthesis process, and maintaining nutrient homeostasis under stress (Bhat et al., 2021; David et al., 2024). At the molecular level, SiNP interacts with various plant biomolecules, including proteins, enzymes, phytohormones, and transcription factors, modulating their activities. Moreover, SiNPs influences signaling pathways, upregulating/downregulating stress-responsive gene expression. SiNPs have also emerged as an efficient nanocarriers for the delivery of nucleic acids, including DNA and RNA, as well as genome-editing platforms such as CRISPR/Cas systems. Recent studies evidently highlighted their ability to enable targeted gene transfer, enhance stress resilience, and support DNA-free genome editing approaches in economically important crop species (Din et al., 2026).

### 6.1 Oxidative stress and biosynthesized SiNPs

The stress stimuli trigger over-production of ROS in plants, surpassing their defense, inducing oxidative damage, which leads to poor plant growth and development, premature senescence, and even plant death. SiNPs alleviate oxidative damage by upregulating the expression of antioxidative defense-related genes. Rapeseed exposed to Cd stress, when treated with biosynthesized SiNPs, upregulated activities of antioxidant enzymes viz., SOD, CAT, APX, and POD by 19.1%, 14.4%, 33.8%, and 33.4%, respectively, which mitigated damage to the ultrastructure of the leaf and oxidative stress (Ahmed et al., 2023). Blueberry plants grown under hypoxic conditions showed a reduction in oxidative and osmotic damage on treatment with SiNPs. The application of SiNPs upregulated the enzymatic and non-enzymatic antioxidant levels along with osmoprotectants, which reduced lipid peroxidation and improved nutrient uptake of Potassium (K), Nitrogen (N), Phosphorus (P), and Zinc (Zn), while reducing Iron (Fe) and Manganese (Mn) content below toxic levels (Iqbal et al., 2021). Analogously, As toxicity (50µM) in *Brassica juncea* was alleviated due to enhanced activity of antioxidants and nutrient homeostasis mediated by 200 ppm SiNPs, diminishing H_2_O_2_ and malondialdehyde (MDA) content by 41% and 39%, respectively (Faisal et al., 2025).

### 6.2 Improving the photosynthetic activity of plants

The photosynthetic machinery suffers damage on exposure to abiotic stresses due to structural and functional changes to chlorophyll pigments, thylakoid membrane, photosynthetic electron transport chain, stomatal conductance, and gaseous exchange (Khan et al., 2024). A decline in the photosynthetic efficiency eventually reduces plant growth and productivity. SiNPs can stimulate the photosynthetic machinery of plants under stress by improving chlorophyll content, the availability of elements like N —a crucial constituent of chlorophyll structure, and the expression of genes responsible for the chlorophyll synthesis (Sami et al., 2020; Mukarram et al., 2022). For instance, SiNP was reported to improve the PS I and II activities, stomatal conductance, Rubisco enzyme activity (due to increased production of the enzyme, reduced transportation of Pb, and improved antioxidants defense facilitated by Si), and chloroplast ultrastructure in *Coriandrum sativum* under lead stress (Fatemi et al., 2020). Analogously, Cd^2+^ stress has been documented to affect the photosynthetic activity and Rubisco enzyme in plants. Rubisco enzyme contain Mg^2+^ ion in its structure that act as a cofactor of carboxylation reaction, which can be substituted by the Cd, affecting structure and activity of the enzyme (Mukarram et al., 2024). SiNPs can reduce Cd-induced toxicity in plants by immobilizing Cd in soil, reducing absorption by plants (Cao et al., 2020), forming a complex with Cd ions, leading to reduced transport and accumulation, and/or by improving antioxidant defense to mitigate oxidative stress (Mukarram et al., 2024), eventually preventing Rubisco degradation. The combined salt and low temperature stress can significantly reduce Rubisco activity in plants (cotton seedlings), due to increased Rubisco exclusion through chloroplast protrusions, diminishing photosynthesis. However, application of Si can restore Rubisco activity, thereby ameliorating stress-induced impact on photosynthetic activity (Liang et al., 2023). SiNPs mediated protection to structure and function of the most crucial cell organelle–the chloroplast, not only imparts stress tolerance, but also improves plant growth and productivity.

### 6.3 Upregulation of gene expression through SiNPs-mediated signaling under stress

SiNP can induce changes in transcriptional programming by upregulating the expression of genes related to antioxidant synthesis, osmolyte production, metal chelation, metal sequestration, and metal transporters to manage stress in plants and improve growth (**Table 2**). For instance, SiNPs downregulate or inhibit the expression of genes encoding uptake of metals like Cd and As (Mukarram et al., 2024). In *Oryza sativa* application of SiNPs downregulated *OsLCT1* and *OsNramp5* genes that partake in Cd uptake and translocation, respectively (Cui et al., 2017) and inhibited *OsLSi1* and *OsLSi2* involved in As uptake (Cui et al., 2020). Application of SiNPs at 60 ppm upregulated expression of four drought-responsive genes i.e., *TaABC1*, *Wdhn13*, *CHP*, and *EXP2* in wheat imparting tolerance to drought stress (Boora et al., 2023). SiNPs also induces a transient ROS burst in plants under stress (Xu et al., 2025), which mediates signal transduction by activating mitogen-activated protein kinase (MAPK), Ca^2+^, nitric oxide (NO), and/or phytohormone signaling pathways such as jasmonic acid (JA), salicylic acid (SA), and abscisic acid (ABA) (Miao et al., 2025) that activate specific TFs including WRKY and DREB, upregulating/downregulating stress-responsive genes such as heat shock proteins (HSP). For instance, application of SiNPs to *Chenopodium quinoa* exposed to Pb stress, alleviated the stress by upregulating RBOH (Respiratory Burst Oxidase Homolog) gene expression, which enhanced the activity of NADPH (Nicotinamide Adenine Dinucleotide Phosphate) oxidase, leading to H_2_O_2_ burst that triggered signaling pathway, upregulating PCS genes and prompted induced systemic acquired acclimatization (Karimi-Baram et al., 2024). Miao et al. (2025) hypothesized the mechanism of SiNPs induced signaling in plants through the absorption of antioxidants and proteins on the surface of SiNPs, forming protein coronas, which elevate the concentration of ROS in the apoplast. Increased apoplastic ROS allow their movement into the cytoplasm via aquaporins and mediates the SA signaling pathway, leading to upregulation of defense response in plants under stress (Miao et al., 2025). Furthermore, the direct interaction of SiNPs with the signaling molecules like PLCP-Zip1, through adsorption on the surface of NPs, induces conformational changes in PLCP, improving or suppressing their proteolytic activities, which in turn activate or inhibit the downstream SA signaling (Miao et al., 2025). Despite multiple efforts, critical knowledge gaps still exist on biosynthesized SiNPs-mediated signaling pathways and stress response activation in plants at the molecular level. More extensive studies elucidating the molecular pathways are therefore pressingly needed.

**Table 2:** Silicon nanoparticles–mediated upregulation of genes expression in plants to improve stress tolerance and growth.

Plant	Stress	Molecular mechanism	Reference
Rice	Salinity	SiNPs upregulated expression of Si transporter genes–*Ls1* and *Ls2* by activating Jasmonic acid signaling pathway. Increased activity of Si transporters promoted Si uptake, which further activated antioxidant and osmolyte production and defense	Abdel-Haliem et al., 2017
Lentils (*Lens culinaris*)	Salinity	Trehalose-coated SiNPs reduced ROS level and oxidative damage, improved water and ionic balance, chlorophyll content by enhancing expression of ion transporters, phytohormones, redox enzymes, photosynthetic genes, and activated glycolysis, MAP kinase, ABA signaling pathways	Sarkar et al., 2026
Strawberry	Salinity	SiNP along with methyl jasmonate increased expression of genes-*DREB*, *cAPX*, *MnSOD*, imparting tolerance to stress and improving plant growth	Moradi et al., 2022
*Ehretia macrophylla*	Drought	Controlled expression of LOX and other redox-related genes, genes involved in auxin and MAPK signaling pathway and metabolism of fatty acids and α-linolenic acids	Chen et al., 2024
Rice	Cadmium	Reduced Cd accumulation and toxicity, improve biochemical and growth parameters, antioxidant level and increased expression of *OsNramp5*, *OsHMA3*, *OsSOD-Cu/Zn*, *OsCATA*, *OsCATB*, and *OsAPX1*	Jalil et al., 2023
*Brassica napus*	Chromium	Reduced translocation of Cr to shoot, abridged ROS content and oxidative stress by enhancing antioxidant defense and upregulated gene expression *BnPsbB*, PsaA, *NADPH-OX*, stress signaling genes—*MAP6* and *MYC2*, and detoxification pathways related genes	Batool et al., 2025

## 7 Environmental fate and toxicity of SiNPs

Toxicity of nanoparticles to living organisms viz., plants, microorganisms, animals, and human being, is a major concern due to the expanding horizon of nanoparticles use in researches, product formulations, and commercial use. The NPs once released to the environment, tend to migrate toward adjacent ecosystems, thereby distributing to larger areas and increasing potential risk of ecotoxicity. Presence of NP, such as SiNP in agricultural soil/water can absorbed and accumulate in plants, increasing chances of food chain contamination and human health hazards owing to bioaccumulation and biomagnification.

Silicon nanoparticles occurring in soil can be absorbed by the plants, owing to small size and high absorption capacity (Bhat et al., 2021) and may induce toxicity. The experimental investigation has reported that the influence of SiNPs on plants varies with the species of plants, and shapes, size, charge, and concentration of administered SiNPs. Studies have demonstrated that SiNPs can induce changes in the pH of the growth media, inducing toxic effects (Naidu et al., 2023), while others have reported SiNPs-induced phytotoxicity in a concentration-dependent manner. The SiNPs induce toxicity at cellular, metabolic, and genomic level. For instance, application of 100 µg ml^-1^ SiNPs induced a cytotoxic effect on root meristematic cells of *Allium cepa*, by inducing significant DNA damage (Liman et al., 2020). Similarly, in *Arabidopsis thaliana*, the low concentration of SiNP was reported to improve the root elongation, but at high doses, induced toxicity (Lee et al., 2010). The large size of SiNPs can mediate toxic effect in plants as larger NPs remain restricted to the apoplastic region, triggering the generation of ROS that may induce oxidative stress in plants rather than activating the signaling pathway. Excess application of SiNPs can clog the pores on the cell membrane, restricting uptake of essential nutrients, which hinders plant growth and development (Okeke et al., 2023). SiNPs can stabilizes other toxic nanomaterials in the soil (Okeke et al., 2023). SiNPs have also been seen to reduce the microbial composition and population, and soil fauna in the soil, alleviating organic matter degradation, soil nutrient turnover, and loss of soil structure (Okeke et al., 2023).

Similar to the plants, toxicity of SiNPs has been reported in aquatic organisms and other living beings. Lajmanovich et al., 2018 conducted a study to evaluate the acute toxicity of colloidal SiNPs on amphibian larvae–*Rhinella arenarum*. The results observed were positive, confirming high toxicity in larvae. Similarly, exposure of Zebrafish to increasing dosages of SiNP resulted in reduced hatching rate, increased mortality and cell death, and embryonic malfunction, resulting in persistent effect on larval behavior (Duan et al., 2013). In mice, 70 nm SiNPs found to induce liver injury, and on repeated administration resulted in hepatic fibrosis (Nishimori et al., 2009). Humans might also get exposed to SiNPs though ingestion of contaminated food, which may cause health hazards. So far no study has been reported on SiNP-induced toxicity in human through consumption of contaminated food. However, this is an area of grave concern, as the toxicity of SiNPs, particularly, amorphous SiNPs on organs/systems such as liver, intestine, immune system, cardiovascular system, and reproductive system have been reported through a study on model organisms or culture cell lines (Ding et al., 2023). Therefore, continuous monitoring of SiNP movement in food chain and evaluating threshold level of SiNPs for inducing adverse effect on human health is needed.

## 8 Challenges and future prospects

Biosynthesized SiNPs can improve plant growth under normal and stressed conditions by modulating gene expression, alleviating stress induced oxidative damage, and improving nutrient assimilation, water content, and photosynthetic activity. Moreover, biological synthesis approach assist industrial and agricultural biowaste management. Being a potential candidate for plant growth, large-scale and commercial application of SiNPs are restricted due to the lack of a detailed molecular mechanism of biosynthesized SiNPs-induced positive as well as negative effects on plants, health risk due to contamination of the food chain, and fate and transportation in environmental matrices. Application of nanoparticles has been known to be associated with the risk of ecotoxicity owing to their small size (Amir et al., 2024a, Amir et al., 2024b). This restrict their commercialization and large-scale on-field applications.

The synthesis of SiNPs via biological approach is sustainable, environmentally benign, and help in managing agro-waste. However, large-scale production faces constraint due to the intensive use of resource like plant and microbial material and techniques like ultracentrifugation and lyophilization for downstream processing, which increases the cost of production (Li et al., 2025), and the price of the product. Additional, green synthesis approach has low silica conversion efficiency and even the silica extraction consistency also varies batch-to-batch, resulting in nanoparticle formation with poor structural consistencies (Li et al., 2025). This further limit the large-scale commercial production and even performance in fields. Thus, the long-term stability and performance of biosynthesized SiNPs in field conditions is not well evaluated. Thus, reliability on their efficiency and increasing scalability is under question. Strict regulatory norms and high cost of application compared to conventional agrochemicals and bulk counterpart are other factors restricting their large-scale commercial application.

Biosynthesized silicon nanoparticles are excellent bioengineered products that have potential to revolutionize the crop cultivation practices, especially under abiotic stresses. Owing to certain limitations, presently SiNPs lie at the back stage in agricultural sector, however, extensive studies, focused researches and use of advanced tools and techniques, could assist in unlocking the potential of SiNPs in food production. Therefore, future studies should concentrate on elucidating detailed molecular mechanism displayed by bioengineered SiNPs’ in plants to improve growth and mediate stress tolerance. Additionally, assessing the change in SiNPs actions owing to factors such as exposure to abiotic stress(es)–singly or simultaneously, plant species, and morphological attributes, sizes and doses of SiNPs, can be achieved through integration of system biology and redox biology. Moreover, integration of nano-enabled agriculture with artificial intelligence (AI) and machine learning (ML) assists in determining the fate, efficiency, and biological output of SiNPs under variable environmental conditions, aid in optimizing most potential method of SiNPs synthesis and facilitate effective decision-making. This will aid in the innovation leading to the development of more effective SiNPs and enables the improvisation in guidelines for on-field deployment, based on the empirical evidences. Studies must be conducted to formulate SiNPs having least or no ecotoxicity by increasing rate of photodegradation in soil and modifying structure to reduce migration to nearby ecosystems. For instance, Sharma et al. (2020) in their study reported the ability of the soil sample to immobilize the surface functionalized silicon nanoparticles, thereby reducing their toxicity in environment.

Studies have shown that the integration of SiNPs with rhizospheric microorganisms can significantly aid in plant growth and crop yield, while increasing tolerance to abiotic stresses. Therefore, future research should concentrate on formulating nano–microbial inoculants using biosynthesized SiNPs and beneficial microorganisms, displaying synergistic effect. Standardizing the dosage of formulated products, assessing consistency in efficacy across plant diversity under multiple stresses and field conditions is also needed to be evaluated. Use of agricultural and industrial waste for bioengineering of silicon nanoparticles is one of the most interesting and pressingly needed option at present era. This approach not only helps formulating nanoparticles in resonance to environmental sanity but facilitate in upcycling the huge volume of waste sustainably, encouraging circular bioeconomy. As silica extraction using biological material result in low output, thus, more technologically-driven studies should be conducted in future to maximize the silica extraction, making the process and the nanoparticle formulation via biological approach, cost- effective.

## 9 Conclusion

Bioengineered silicon nanoparticles approach possess multiple advantages over conventionally synthesized NPs and bulk silicon, such as small size, large surface area, biocompatibility, better absorption rates in plants, sustainable production with low chemical and energy inputs, and encourages circular bioeconomy, which makes them desirable for agricultural use. Treatment of plants and soil with SiNPs improve soil structure and function, promote plant growth and development, and impart tolerance to several abiotic stresses by strengthening cell wall, preserving structure and functions of the photosynthetic apparatus, ameliorating oxidative damage, increasing water use efficiency and nutrient assimilation, and stimulating beneficial soil microbial population and diversity. These beneficial physiological and biochemical effects are facilitated by SiNPs at molecular level by eliciting signaling pathways such as mitogen-activated protein kinase and phytohormones signaling pathways, which mediate upregulation of stress-responsive genes. Despite several advantages, limited understanding of the precise molecular mechanism underlying SiNPs-mediated tolerance in different plant genera under different abiotic stresses, long-term efficiency in field conditions, and environmental fate continues to hamper their large-scale field deployment. To promote the growth and yield of crop plants exposed to abiotic stresses, effective application of SiNPs are crucial. Therefore, more scientific studies with viable field applications must be undertaken. Additionally, new avenues must be prospected to improve the production, commercialization, and application of biogenic SiNPs.

Some future recommendations to promote sustainable and effective application of SiNPs in agriculture are as follows:

- A comprehensive investigation integrating system biology and redox biology with nanotechnology is required to elucidate the plant growth–improving and stress alleviating mechanisms in plants at the genomic level.
- Integration of AI and ML tools with nano-enabled agricultural activities should be encouraged to evaluate the NP synthesis approaches, their environmental fate, and efficacy under variable environmental conditions. This approach facilitates effective decision-making, ensuring environmental sustainability and food security.
- Encouraging on-field studies to evaluate the effect of persisting environmental conditions on the competence of biosynthesized SiNPs.
- Evaluating the efficacy of biosynthesized SiNPs with varying shapes, sizes, and concentrations on plants belonging to different families under abiotic stresses.
- Undertaking studies to assess the transport and accumulation of biosynthesized SiNPs to adjacent ecosystems and food chain and empirically analyzing their toxicity across biota, including human beings.

## References

[B1] Abdel-Haliem M. E. Hegazy H. S. Hassan N. S. Naguib D. M. (2017). Effect of silica ions and nano silica on rice plants under salinity stress. Ecol. Eng 99, 282–289. doi: 10.1016/j.ecoleng.2016.11.060 38826717

[B2] Abdelhamid M. A. Khalifa H. O. Ki M. R. Pack S. P. (2024). Nanoengineered silica-based biomaterials for regenerative medicine. Int. J. Mol. Sci. 25, 6125. doi: 10.3390/ijms25116125 38892312 PMC11172759

[B3] Adebisi J. A. Agunsoye J. O. Ahmed I. I. Bello S. A. Haris M. Ramakokovhu M. M. . (2021). Production of silicon nanoparticles from selected agricultural wastes. Mater. Today Proc. 38, 669–674. doi: 10.1016/j.matpr.2020.03.658 38826717

[B4] Ahmad R. Zaheer S. H. Ismail S. (1992). Role of silicon in salt tolerance of wheat (Triticum aestivum L.). Plant Sci. 85, 43–50. doi: 10.1016/0168-9452(92)90092-z

[B5] Ahmed T. Masood H. A. Noman M. Al-Huqail A. A. Alghanem S. M. Khan M. M. . (2023). Biogenic silicon nanoparticles mitigate cadmium (Cd) toxicity in rapeseed (Brassica napus L.) by modulating the cellular oxidative stress metabolism and reducing Cd translocation. J. Hazard Mater. 459, 132070. doi: 10.1016/j.jhazmat.2023.132070 37478591

[B6] Akhayere E. Kavaz D. Vaseashta A. (2022). Efficacy studies of silica nanoparticles synthesized using agricultural waste for mitigating waterborne contaminants. Appl. Sci. 12, 9279. doi: 10.3390/app12189279 30654563

[B7] Alsaeedi A. H. Elgarawany M. M. El-Ramady H. Alshaal T. Al-Otaibi A. O. A. (2019). Application of silica nanoparticles induces seed germination and growth of cucumber (Cucumis sativus). Met Environ. Arid Land Agric. Sci. 28, 57–68. doi: 10.4197/Met.28-1.6

[B8] Alsamadany H. Alharby H. F. Ahmad Z. Al-Zahrani H. S. Alzahrani Y. M. Almaghamsi A. (2024). Improving alkaline stress tolerance in maize through seed priming with silicon nanoparticles: a comprehensive investigation of growth, photosynthetic pigments, antioxidants, and ion balance. Silicon 16, 2233–2244. doi: 10.1007/s12633-023-02833-5 30311153

[B9] Altaf M. M. Yi H. Bashir S. Abbasi S. S. Anwar M. Alsahli A. A. . (2024). Mitigating chromium stress in tomato plants using green-silicone nanoparticles: Enhancing cellular oxidative stress management and chromium reduction. Sci. Hortic. 338, 113635. doi: 10.1016/j.scienta.2024.113635 38826717

[B10] Amir M. Raheem A. Kumar A. Jalil S. U. Shadab M. Ansari N. G. . (2024a). Role of phytofabricated gold nanoparticles for enhancing sustainable Spinacia oleracea L. production. South Afr J. Bot. 166, 386–397. doi: 10.1016/j.sajb.2024.01.028 38826717

[B11] Amir M. Raheem A. Yadav P. Kumar V. Tewari R. K. Jalil S. U. . (2024b). Phytofabricated gold nanoparticles as modulators of salt stress responses in spinach: implications for redox homeostasis, biochemical and physiological adaptation. Front. Plant Sci. 15, 1408642. doi: 10.3389/fpls.2024.1408642 38957605 PMC11217327

[B12] Amir M. M. Abdul Raheem A. Jalil S. U. Danish M. Ansari M. I. (2025). Green synthesized silver quantum dot mitigates arsenic translocation in spinach via glutathione-driven redox balance and modulation of stomatal-transpiration dynamics. Plant Stress 18, 101138. doi: 10.1016/j.stress.2025.101138 38826717

[B13] Ansari M. I. Lin T. P. (2010). Molecular analysis of dehydration in plants. Int. Res. J. Plant Sci. 1, 21–25.

[B14] Antu U. B. Roy T. K. Roshid M. M. Mitu P. R. Barman M. K. Tazry J. . (2025). Perspective of nanocellulose production, processing, and application in sustainable agriculture and soil fertility enhancement: A potential review. Int. J. Biol. Macromol 303, 140570. doi: 10.1016/j.ijbiomac.2025.140570 39904432

[B15] Athinarayanan J. Jaafari S. A. A. H. Periasamy V. S. Almanaa T. N. A. Alshatwi A. A. (2020). Fabrication of biogenic silica nanostructures from Sorghum bicolor leaves for food industry applications. Silicon 12, 2829–2836. doi: 10.1007/s12633-020-00379-4 30311153

[B16] Azim Z. Singh N. B. Singh A. Amist N. Niharika Khare S. . (2023). A review summarizing uptake, translocation and accumulation of nanoparticles within the plants: current status and future prospectus. J. Plant Biochem. Biotechnol. 32, 211–224. doi: 10.1007/s13562-022-00800-6 30311153

[B17] Babu R. H. Yugandhar P. Savithramma N. (2018). Synthesis, characterization and antimicrobial studies of bio silica nanoparticles prepared from Cynodon dactylon L.: a green approach. Bull. Mater. Sci. 41, 65. doi: 10.1007/s12034-018-1584-4 30311153

[B18] Baio J. E. Zane A. Jaeger V. Roehrich A. M. Lutz H. Pfaendtner J. . (2014). Diatom mimics: directing the formation of biosilica nanoparticles by controlled folding of lysine-leucine peptides. J. Am. Chem. Soc 136, 15134–15137. doi: 10.1021/ja5078238 25285787 PMC4608251

[B19] Bansal V. Rautaray D. Bharde A. Ahire K. Sanyal A. Ahmad A. . (2005). Fungus-mediated biosynthesis of silica and titania particles. J. Mater. Chem. 15, 2583–2589. doi: 10.1039/b503008k

[B20] Batool I. Hu Y. Zhang K. Hannan F. Sun Y. Qin T. . (2025). Integrated transcriptomic and physiological analyses elucidate the role of silicon nanoparticles in chromium detoxification in Brassica napus. Plant Stress 19, 101192. doi: 10.1016/j.stress.2025.101192 38826717

[B21] Bhardwaj S. Sharma D. Singh S. Ramamurthy P. C. Verma T. Pujari M. . (2023). Physiological and molecular insights into the role of silicon in improving plant performance under abiotic stresses. Plant Soil 486, 25–43. doi: 10.1007/s11104-022-05395-4 30311153

[B22] Bhat J. A. Rajora N. Raturi G. Sharma S. Dhiman P. Sanand S. . (2021). Silicon nanoparticles (SiNPs) in sustainable agriculture: major emphasis on the practicality, efficacy and concerns. Nanoscale Adv. 3, 4019–4028. doi: 10.1039/d1na00233c 36132841 PMC9419652

[B23] Boora R. Sheoran P. Rani N. Kumari S. Thakur R. Grewal S. (2023). Biosynthesized silica nanoparticles (Si NPs) helps in mitigating drought stress in wheat through physiological changes and upregulation of stress genes. Silicon 15, 5565–5577. doi: 10.1007/s12633-023-02439-x 30311153

[B24] Cai Y. Liu Z. Wang H. Meng H. Cao Y. (2024). Mesoporous silica nanoparticles mediate SiRNA delivery for long‐term multi‐gene silencing in intact plants. Adv. Sci. 11, 2301358. doi: 10.1002/advs.202301358 38145358 PMC10916655

[B25] Cao P. Qiu K. Zou X. Lian M. Liu P. Niu L. . (2020). Mercapto propyltrimethoxysilane-and ferrous sulfate-modified nano-silica for immobilization of lead and cadmium as well as arsenic in heavy metal-contaminated soil. Environ. pollut. 266, 115152. doi: 10.1016/j.envpol.2020.115152 32702603

[B26] Cao Y. Bolisetty S. Wolfisberg G. Adamcik J. Mezzenga R. (2019). Amyloid fibril-directed synthesis of silica core–shell nanofilaments, gels, and aerogels. Proc. Natl. Acad. Sci. 116, 4012–4017. doi: 10.1073/pnas.1819640116 30782823 PMC6410827

[B27] Chang H. Kim J. Rho W. Y. Pham X. H. Lee J. H. Lee S. H. . (2021). Silica nanoparticles. Nanotechnol For. Bioapplications 1309, 41–65. doi: 10.1007/978-981-33-6158-4_3 33782868

[B28] Chen M. Jiao S. Q. Xie L. Geng X. Qi S. Fan J. . (2024). Integrated physiological, transcriptomic, and metabolomic analyses of drought stress alleviation in Ehretia macrophylla Wall. seedlings by SiO2 NPs (silica nanoparticles). Front. Plant Sci. 15, 1260140. doi: 10.3389/fpls.2024.1260140 38371410 PMC10869631

[B29] Costa M. G. de Mello Prado R. Palaretti L. F. de Souza Júnior J. P. (2024). The effect of abiotic stresses on plant C: N: P homeostasis and their mitigation by silicon. Crop J. 12, 340–353. doi: 10.1016/j.cj.2023.11.012 38826717

[B30] Cui J. Li Y. Jin Q. Li F. (2020). Silica nanoparticles inhibit arsenic uptake into rice suspension cells via improving pectin synthesis and the mechanical force of the cell wall. Environ. Sci. Nano 7, 162–171. doi: 10.1201/9781003317395-80

[B31] Cui J. Liu T. Li F. Yi J. Liu C. Yu H. (2017). Silica nanoparticles alleviate cadmium toxicity in rice cells: mechanisms and size effects. Environ. pollut. 228, 363–369. doi: 10.1016/j.envpol.2017.05.014 28551566

[B32] David O. A. Labulo A. H. Hassan I. Olawuni I. Oseghale C. O. Terna A. D. . (2024). Complexation and immobilization of arsenic in maize using green synthesized silicon nanoparticles (SiNPs). Sci. Rep. 14, 6176. doi: 10.1038/s41598-024-56924-3 38486015 PMC10940286

[B33] Din T. M. U. Hurrah I. M. Banday L. Mandal S. (2026). “ Silicon nanoparticles in plant genetic engineering,” in Nano-Delivering for Plant Genetic Engineering. Smart Nanomaterials Technology. Ed. Chen J. T. ( Springer, Singapore).

[B34] Ding R. Li Y. Yu Y. Sun Z. Duan J. (2023). Prospects and hazards of silica nanoparticles: Biological impacts and implicated mechanisms. Biotechnol. Adv. 69, 108277. doi: 10.1016/j.bioteChadv.2023.108277 37923235

[B35] Dorairaj D. Govender N. Zakaria S. Wickneswari R. (2022). Green synthesis and characterization of UKMRC-8 rice husk-derived mesoporous silica nanoparticle for agricultural application. Sci. Rep. 12, 20162. doi: 10.1038/s41598-022-24484-z 36424408 PMC9691743

[B36] Dorjee L. Wangmu T. (2023). Role of Silicon in inducing fungal disease resistance in Rice. Sci. World 3, 2817–2824. doi: 10.5281/zenodo.10072950

[B37] Duan J. Yu Y. Shi H. Tian L. Guo C. Huang P. . (2013). Toxic effects of silica nanoparticles on zebrafish embryos and larvae. PloS One 8, e74606. doi: 10.1371/journal.pone.0074606 24058598 PMC3776836

[B38] El-Aziz M. A. A. Elbagory M. Arafat A. A. Aboelsoud H. M. El-Nahrawy S. Khalifa T. H. . (2025). Evaluating the impact of nano-silica and silica hydrogel amendments on soil water retention and crop yield in rice and clover under variable irrigation conditions. Agronomy 15, 652. doi: 10.3390/agronomy15030652 30654563

[B39] El-Saadony M. T. Desoky E. S. M. Saad A. M. Eid R. S. Selem E. Elrys A. S. (2021). Biological silicon nanoparticles improve Phaseolus vulgaris L. yield and minimize its contaminant contents on a heavy metals-contaminated saline soil. J. Environ. Sci. 106, 1–14. doi: 10.1016/j.jes.2021.01.012 34210425

[B40] Emamverdian A. Ding Y. Mokhberdoran F. Xie Y. Zheng X. Wang Y. (2020). Silicon dioxide nanoparticles improve plant growth by enhancing antioxidant enzyme capacity in bamboo (Pleioblastus pygmaeus) under lead toxicity. Trees 34, 469–481. doi: 10.1007/s00468-019-01929-z 30311153

[B41] Estevez M. Vargas S. Castano V. M. Rodriguez R. (2009). Silica nano-particles produced by worms through a bio-digestion process of rice husk. J. Non-Cryst Solids 355, 844–850. doi: 10.1016/j.jnoncrysol.2009.04.011 38826717

[B42] Faisal M. Bilmez Ozcinar A. Karadeniz E. Faizan M. Sultan H. Alatar A. A. (2025). Supplementation of silicon oxide nanoparticles mitigates the damaging effects of arsenic stress on photosynthesis, antioxidant mechanism and nitrogen metabolism in Brassica juncea. Sci. Rep. 15, 21476. doi: 10.1038/s41598-025-04553-9 40595861 PMC12218454

[B43] Fatemi H. Pour B. E. Rizwan M. (2020). Isolation and characterization of lead (Pb) resistant microbes and their combined use with silicon nanoparticles improved the growth, photosynthesis and antioxidant capacity of coriander (Coriandrum sativum L.) under Pb stress. Environ. pollut. 266, 114982. doi: 10.1016/j.envpol.2020.114982 32650299

[B44] Ghouri F. Sarwar S. Sun L. Riaz M. Haider F. U. Ashraf H. . (2024). Silicon and iron nanoparticles protect rice against lead (Pb) stress by improving oxidative tolerance and minimizing Pb uptake. Sci. Rep. 14, 5986. doi: 10.1038/s41598-024-55810-2 38472251 PMC10933412

[B45] Goswami P. Mathur J. (2022). Application of agro-waste-mediated silica nanoparticles to sustainable agriculture. Bioresour Bioprocess 9, 9. doi: 10.1186/s40643-022-00496-5 38647762 PMC10992809

[B46] Goswami P. Mathur J. Srivastava N. (2022). Silica nanoparticles as novel sustainable approach for plant growth and crop protection. Heliyon 8. doi: 10.1016/j.heliyon.2022.e09908 35847613 PMC9284391

[B47] Haghighi M. Pessarakli M. (2013). Influence of silicon and nano-silicon on salinity tolerance of cherry tomatoes (Solanum lycopersicum L.) at early growth stage. Sci. Hortic. 161, 111–117. doi: 10.1016/j.scienta.2013.06.034 38826717

[B48] Harish V. Ansari M. M. Tewari D. Gaur M. Yadav A. B. García-Betancourt M. L. . (2022). Nanoparticle and nanostructure synthesis and controlled growth methods. Nanomaterials 12, 3226. doi: 10.3390/nano12183226 36145012 PMC9503496

[B49] Hatami M. Khanizadeh P. Bovand F. Aghaee A. (2021). Silicon nanoparticle-mediated seed priming and Pseudomonas spp. inoculation augment growth, physiology and antioxidant metabolic status in Melissa officinalis L. plants. Ind. Crops Prod. 162, 113238. doi: 10.1016/j.indcrop.2021.113238 38826717

[B50] Huang Q. Ayyaz A. Farooq M. A. Zhang K. Chen W. Hannan F. . (2024). Silicon dioxide nanoparticles enhance plant growth, photosynthetic performance, and antioxidants defence machinery through suppressing chromium uptake in Brassica napus L. Environ. pollut. 342, 123013. doi: 10.1016/j.envpol.2023.123013 38012966

[B51] Iqbal M. S. Singh A. K. Singh S. P. Ansari M. I. (2020). “ Nanoparticles and plant interaction with respect to stress response,” in Nanomaterials and Environmental Biotechnology ( Springer International Publishing, Cham), 1–15.

[B52] Iqbal Z. Sarkhosh A. Balal R. M. Rauf S. Khan N. Altaf M. A. . (2021). Silicon nanoparticles mitigate hypoxia-induced oxidative damage by improving antioxidants activities and concentration of osmolytes in southern highbush blueberry plants. Agronomy 11, 2143. doi: 10.3390/agronomy11112143 30654563

[B53] Jabeen N. Maqbool Q. Sajjad S. Minhas A. Younas U. Anwaar S. . (2017). Biosynthesis and characterisation of nano‐silica as potential system for carrying streptomycin at nano‐scale drug delivery. IET Nanobiotechnol 11, 557–561. doi: 10.1049/iet-nbt.2016.0106 28745289 PMC8676573

[B54] Jackson E. Ferrari M. Cuestas-Ayllon C. Fernández-Pacheco R. Perez-Carvajal J. de la Fuente J. M. . (2015). Protein-templated biomimetic silica nanoparticles. Langmuir 31, 3687–3695. doi: 10.1021/la504978r 25741589

[B55] Jalil S. U. Ansari M. I. (2019). “ Nanoparticles and abiotic stress tolerance in plants: synthesis, action, and signaling mechanisms,” in Plant Signaling Molecules, 549–561.

[B56] Jalil S. U. Ansari M. I. (2021). “ Role of nanomaterials in regulating reactive species as a signaling molecule of abiotic stress in plants,” in Nanobiotechnology, Springer Publisher, 291–304. doi: 10.1007/978-3-030-73606-4_12

[B57] Jalil S. Nazir M. M. Al-Huqail A. A. Ali B. Al-Qthanin R. N. Asad M. A. . (2023). Silicon nanoparticles alleviate cadmium toxicity in rice (Oryza sativa L.) by modulating the nutritional profile and triggering stress-responsive genetic mechanisms. Ecotoxicol Environ. Saf. 268, 115699. doi: 10.1016/j.ecoenv.2023.115699 37979353

[B58] Jalil S. U. Zahera M. Khan M. S. Ansari M. I. (2019). Biochemical synthesis of gold nanoparticles from leaf protein of Nicotiana tabacum L. cv. xanthi and their physiological, developmental, and ROS scavenging responses on tobacco plant under stress conditions. IET Nanobiotechnol. Woodhead Publishing (Elsevier). 13, 23–29. doi: 10.1049/iet-nbt.2018.5148 30964033 PMC8676148

[B59] Janmohammadi M. Sabaghnia N. (2015). Effect of pre-sowing seed treatments with silicon nanoparticles on germinability of sunflower (Helianthus annuus). Bot. Lith. 21, 13–21. doi: 10.1515/botlit-2015-0002 31755547

[B60] Karimi-Baram A. Amooaghaie R. Ghorbanpour M. Ahadi A. (2024). RBOH-dependent H2O2 burst induced by silicon dioxide nanoparticles establishes systemic acquired acclimation in quinoa under lead toxicity. Sci. Hortic. 335, 113366. doi: 10.1016/j.scienta.2024.113366 38826717

[B61] Kaur H. Greger M. (2019). A review on Si uptake and transport system. Plants 8, 81. doi: 10.3390/plants8040081 30934978 PMC6524041

[B62] Khan I. Awan S. A. Rizwan M. Huizhi W. Ulhassan Z. Xie W. (2024). Silicon nanoparticles improved the osmolyte production, antioxidant defense system, and phytohormone regulation in Elymus sibiricus (L.) under drought and salt stress. Environ. Sci. pollut. Res. 31, 8985–8999. doi: 10.1007/s11356-023-31730-y 38183551

[B63] Lajmanovich R. C. Peltzer P. M. Martinuzzi C. S. Attademo A. M. Colussi C. L. Basso A. (2018). Acute toxicity of colloidal silicon dioxide nanoparticles on amphibian larvae: emerging environmental concern. Int. J. Environ. Res. 12, 269–278. doi: 10.1007/s41742-018-0089-8 30311153

[B64] Lee C. W. Mahendra S. Zodrow K. Li D. Tsai Y. C. Braam J. . (2010). Developmental phytotoxicity of metal oxide nanoparticles to Arabidopsis thaliana. Environ. Toxicol. Chem. 29, 669–675. doi: 10.1002/etc.58 20821493

[B65] Li S. Gu X. Wang S. Wang L. Lin Y. Liang X. . (2024). Rhamnolipid modified silica nanoparticles control rice blast disease by enhancing antifungal activity *in vivo* and antioxidant defense system of rice (Oryza sativa L.). ACS Appl. Materials Interfaces 17, 1792–1802. doi: 10.1021/acsami.4c11833 39704214

[B66] Li F. Hou Y. Chen L. Qiu Y. (2025). Advances in silica nanoparticles for agricultural applications and biosynthesis. Advanced Biotechnol. 3, 1–14. doi: 10.1007/s44307-025-00067-7 40289240 PMC12034607

[B67] Lian M. Wang L. Feng Q. Niu L. Zhao Z. Wang P. . (2021). Thiol-functionalized nano-silica for in-situ remediation of Pb, Cd, Cu contaminated soils and improving soil environment. Environ. pollut. 280, 116879. doi: 10.1016/j.envpol.2021.116879 33774545

[B68] Liang X. Liao Q. Guo P. Yang Z. Kang S. Du T. . (2026). Silicon nanoparticles enhance maize yield and water productivity via regulating photosynthesis and canopy structure under mild regulated deficit irrigation. Front. Plant Sci. 16, 1691443. doi: 10.3389/fpls.2025.169144 41567404 PMC12816220

[B69] Liang Y. Liu H. Fu Y. Li P. Li S. Gao Y. (2023). Regulatory effects of silicon nanoparticles on the growth and photosynthesis of cotton seedlings under salt and low-temperature dual stress. BMC Plant Biol. 23, 504. doi: 10.1186/s12870-023-04509-z 37864143 PMC10589941

[B70] Liang Y. Liu H. Zhang Y. Li P. Fu Y. Li S. . (2024). Exogenous application of silica nanoparticles mitigates combined salt and low-temperature stress in cotton seedlings by improving the K+/Na+ ratio and antioxidant defense. Plant Stress 14, 100597. doi: 10.1016/j.stress.2024.100597 38826717

[B71] Liman R. Acikbas Y. Ciğerci İ.H. Ali M. M. Kars M. D. (2020). Cytotoxic and genotoxic assessment of silicon dioxide nanoparticles by allium and comet tests. Bull. Environ. Contamination Toxicol. 104, 215–221. doi: 10.1007/s00128-020-02783-3 31932906

[B72] Ma Y. He X. Zhang P. Zhang Z. Ding Y. Zhang J. . (2017). Xylem and phloem based transport of CeO2 nanoparticles in hydroponic cucumber plants. Environ. Sci. Technol. 51, 5215–5221. doi: 10.1021/acs.est.6b05998 28383248

[B73] Malik M. S. Rehman A. Shah I. H. Arif S. Nan K. Yan Y. . (2025). Green synthesized silicon dioxide nanoparticles (SiO2NPs) ameliorated the cadmium toxicity in melon by regulating antioxidant enzymes activity and stress-related genes expression. Environ. pollut. 366, 125459. doi: 10.1016/j.envpol.2024.125459 39644955

[B74] Malpani S. K. Goyal D. (2023). Synthesis, analysis, and multi-faceted applications of solid wastes-derived silica nanoparticles: a comprehensive review, (2010–2022). Environ. Sci. pollut. Res. 30, 28321–28343. doi: 10.1007/s11356-022-23873-1 36331737

[B75] Manimaran G. Duraisamy S. Subramanium T. Rangasamy A. Alagarsamy S. James P. . (2025). Silicon-driven approaches to salinity stress tolerance: Mechanisms, uptake dynamics, and microbial transformations. Plant Stress 16, 100825. doi: 10.1016/j.stress.2025.100825 38826717

[B76] Marzaioli V. Aguilar-Pimentel J. A. Weichenmeier I. Luxenhofer G. Wiemann M. Landsiedel R. . (2014). Surface modifications of silica nanoparticles are crucial for their inert versus proinflammatory and immunomodulatory properties. Int. J. Nanomed 5, 2815–2832. doi: 10.2147/ijn.s57396 24940059 PMC4051720

[B77] Maurya A. K. Rathi S. Saksena Y. P. Awasthi A. Kaushik B. Sheetal (2025). “ Uptake, translocation and consequences of application of Si/SiO2 nanoparticles (SiNPs) in plants,” in Silicon Nanoparticles: Synthesis, Uptake, and Applications, 213–261.

[B78] Miao G. Han J. Han T. (2025). Silicon nanoparticles and apoplastic protein interaction: A hypothesized mechanism for modulating plant growth and immunity. Plants 14, 1630. doi: 10.3390/plants14111630 40508305 PMC12157989

[B79] Mir R. A. Bhat B. A. Yousuf H. Islam S. T. Raza A. Rizvi M. A. . (2022). Multidimensional role of silicon to activate resilient plant growth and to mitigate abiotic stress. Front. Plant Sci. 13, 819658. doi: 10.3389/fpls.2022.819658 35401625 PMC8984490

[B80] Misra V. Mall A. K. Ansari S. A. Raheem A. Tripathi M. K. Ansari M. I. (2023). Silicon as a beneficial nutrient for productivity augmentation and abiotic/biotic stress tolerance in sugarcane. Biocatal Agric. Biotechnol. 54, 102944. doi: 10.1016/j.bcab.2023.102944 38826717

[B81] Moradi P. Vafaee Y. Mozafari A. A. Tahir N. A. R. (2022). Silicon nanoparticles and methyl jasmonate improve physiological response and increase expression of stress-related genes in strawberry cv. Paros under salinity stress. Silicon 14, 10559–10569. doi: 10.1007/s12633-022-01791-8 30311153

[B82] Mostofa M. G. Rahman M. M. Ansary M. M. U. Keya S. S. Abdelrahman M. Miah M. G. . (2021). Silicon in mitigation of abiotic stress-induced oxidative damage in plants. Crit. Rev. Biotechnol. 41, 918–934. doi: 10.1080/07388551.2021.1892582 33784900

[B83] Mukarram M. Ahmad B. Choudhary S. Konôpková A. S. Kurjak D. Khan M. M. A. . (2024). Silicon nanoparticles vs trace elements toxicity: Modus operandi and its omics bases. Front. Plant Sci. 15, 1377964. doi: 10.3389/fpls.2024.1377964 38633451 PMC11021597

[B84] Mukarram M. Khan M. M. A. Corpas F. J. (2021). Silicon nanoparticles elicit an increase in lemongrass (Cymbopogon flexuosus (Steud.) Wats) agronomic parameters with a higher essential oil yield. J. Hazard Mater. 412, 125254. doi: 10.1016/j.jhazmat.2021.125254 33550131

[B85] Mukarram M. Petrik P. Mushtaq Z. Khan M. M. A. Gulfishan M. Lux A. (2022). Silicon nanoparticles in higher plants: Uptake, action, stress tolerance, and crosstalk with phytohormones, antioxidants, and other signalling molecules. Environ. pollut. 310, 119855. doi: 10.1016/j.envpol.2022.119855 35940485

[B86] Naaz S. Sachdev S. Husain R. Pandey V. Ansari M. I. (2023). “ Nanomaterials and nanocomposites: significant uses in plant performance, production, and toxicity response,” in Nanomaterials and Nanocomposites Exposures to Plants: Response, Interaction, Phytotoxicity and Defense Mechanisms ( Springer Nature Singapore, Singapore), 1–18.

[B87] Naidu S. Pandey J. Mishra L. C. Chakraborty A. Roy A. Singh I. K. . (2023). Silicon nanoparticles: Synthesis, uptake and their role in mitigation of biotic stress. Ecotoxicol Environ. Saf. 255, 114783. doi: 10.1016/j.ecoenv.2023.114783 36963184

[B88] Natesan K. Ponmurugan P. Gnanamangai B. M. Manigandan V. Joy S. P. J. Jayakumar C. . (2021). Biosynthesis of silica and copper nanoparticles from Trichoderma, Streptomyces and Pseudomonas spp. evaluated against collar canker and red root-rot disease of tea plants. Arch. Phytopathol. Plant Prot. 54, 56–85. doi: 10.1080/03235408.2020.1817258 37339054

[B89] Nguyen T. K. M. Ki M. R. Son R. G. Kim K. H. Hong J. Pack S. P. (2021). Synthesis of sub-50 nm bio-inspired silica particles using a C-terminal-modified ferritin template with a silica-forming peptide. J. Ind. Eng Chem. 101, 262–269. doi: 10.1016/j.jiec.2021.06.005 38826717

[B90] Nishimori H. Kondoh M. Isoda K. Tsunoda S. I. Tsutsumi Y. Yagi K. (2009). Silica nanoparticles as hepatotoxicants. Eur. J. Pharm. Biopharm 72, 496–501. doi: 10.1016/j.ejpb.2009.02.005 19232391

[B91] Okeke E. S. Nweze E. J. Ezike T. C. Nwuche C. O. Ezeorba T. P. C. Nwankwo C. E. I. (2023). Silicon-based nanoparticles for mitigating the effect of potentially toxic elements and plant stress in agroecosystems: a sustainable pathway towards food security. Sci. Total Environ. 898, 165446. doi: 10.1016/j.scitotenv.2023.165446 37459984

[B92] Panigrahi L. L. Rout G. R. (2025). Mechanistic insights into micro and nanosilicon for environmental stress mitigation in plant. Discover Nano 20, 208. doi: 10.1186/s11671-025-04387-4 41258352 PMC12630478

[B93] Parveen N. U. S. R. A. T. Ashraf M. U. H. A. M. M. A. D. (2010). Role of silicon in mitigating the adverse effects of salt stress on growth and photosynthetic attributes of two maize (Zea mays L.) cultivars grown hydroponically. Pak. J. Bot. 42, 1675–1684.

[B94] Periakaruppan R. N R. D. Abed S. A. Vanathi P. Kumar J. S. (2023). Production of biogenic silica nanoparticles by green chemistry approach and assessment of their physicochemical properties and effects on the germination of sorghum bicolor. Silicon 15, 4309–4316. doi: 10.1007/s12633-023-02348-z 30311153

[B95] Periakaruppan R. S M. P. C P. P R. S G. R. Danaraj J. (2022). Biosynthesis of silica nanoparticles using the leaf extract of Punica granatum and assessment of its antibacterial activities against human pathogens. Appl. Biochem. Biotechnol. 194, 5594–5605. doi: 10.21203/rs.3.rs-1458242/v1 35679016

[B96] Pinedo-Guerrero Z. H. Cadenas-Pliego G. Ortega-Ortiz H. González-Morales S. Benavides-Mendoza A. Valdés-Reyna J. . (2020). Form of silica improves yield, fruit quality and antioxidant defense system of tomato plants under salt stress. Agriculture 10, 367. doi: 10.3390/agriculture10090367 30654563

[B97] Raheem A. Amir M. Yadav P. Singh R. K. Kumar A. Jalil S U. . (2025). Unveiling the potential of Piper betle-mediated gold nanoparticles in augmenting growth and yield of Vigna mungo L. J. Plant Growth Regul. 44, 6581–6596. doi: 10.1007/s00344-025-11845-x 30311153

[B98] Rajput V. D. Minkina T. Feizi M. Kumari A. Khan M. Mandzhieva S. . (2021). Effects of silicon and silicon-based nanoparticles on rhizosphere microbiome, plant stress and growth. Biology 10, 791. doi: 10.3390/biology10080791 34440021 PMC8389584

[B99] Rastogi A. Tripathi D. K. Yadav S. Chauhan D. K. Živčák M. Ghorbanpour M. . (2019). Application of silicon nanoparticles in agriculture. 3 Biotech. 9, 90. doi: 10.1007/s13205-019-1626-7 30800601 PMC6385075

[B100] Raza M. A. S. Zulfiqar B. Iqbal R. Muzamil M. N. Aslam M. U. Muhammad F. . (2023). Morpho-physiological and biochemical response of wheat to various treatments of silicon nano-particles under drought stress conditions. Sci. Rep. 13, 2700. doi: 10.1038/s41598-023-29784-6 36792788 PMC9931706

[B101] Reichinger D. Reithofer M. Hohagen M. Drinic M. Tobias J. Wiedermann U. . (2022). A biomimetic, silaffin R5-based antigen delivery platform. Pharmaceutics 15, 121. doi: 10.3390/pharmaceutics15010121 36678751 PMC9866965

[B102] Roy T. K. Islam M. S. Antu U. B. Sumi M. J. Irin I. J. Hossain S. A. . (2025a). Exogenous application of ZnO nanoparticles (NPs) to palliate salinity induced oxidative stress in lettuce (Lactuca sativa L.) plant. J. Soil Sci. Plant Nutr. 25, 6398–6412. doi: 10.1007/s42729-025-02537-2 30311153

[B103] Roy T. K. Islam M. S. Mahiddin N. A. Hossain S. A. Biswas T. Antu U. B. . (2025b). Application of nanoparticles (NPs) to ameliorate abiotic stress in economically important crop species: a potential review. J. Crop Health 77, 19. doi: 10.1007/s10343-024-01069-6 30311153

[B104] Sachdev S. Ahmad S. (2021). “ Role of nanomaterials in regulating oxidative stress in plants,” in Nanobiotechnology: Mitigation of Abiotic Stress in Plants ( Springer International Publishing, Cham), 305–326.

[B105] Sachdev S. Ansari S. A. Ansari M. I. (2023). “ Oxidative stress triggered damage to cellular biomolecules,” in Reactive Oxygen Species in Plants: The Right Balance ( Springer Nature Singapore, Singapore), 45–59.

[B106] Sachdev S. Ansari S. A. Ansari M. I. Fujita M. Hasanuzzaman M. (2021). Abiotic stress and reactive oxygen species: Generation, signaling, and defense mechanisms. Antioxidants 10, 277. doi: 10.3390/antiox10020277 33670123 PMC7916865

[B107] Sachdev S. Keswani C. Minkina T. Bauddh K. (2025). Mechanisms of microbe-assisted metal tolerance in phytoremediators: A review. Pedosphere 35, 249–263. doi: 10.1016/j.pedsph.2024.09.003 38826717

[B108] Sajida Kashtoh H. Lama Tamang T. Baek K. H. (2025). Recent advances on the individual roles and emerging synergistic effects of plant growth-promoting rhizobacteria and silicon nanoparticles in mitigating salinity stress. Plants 14, 3632. doi: 10.3390/plants14233632 41375342 PMC12694149

[B109] Sami F. Siddiqui H. Hayat S. (2020). “ Impact of silver nanoparticles on plant physiology: a critical review,” in Sustainable Agriculture Reviews 41: Nanotechnology for Plant Growth and Development, 111–127.

[B110] Sankar S. Sharma S. K. Kaur N. Lee B. Kim D. Y. Lee S. . (2016). Biogenerated silica nanoparticles synthesized from sticky, red, and brown rice husk ashes by a chemical method. Ceram Int. 42, 4875–4885. doi: 10.1016/j.ceramint.2015.11.172 38826717

[B111] Sarkar M. M. Ghosh R. Roy S. (2026). Integrative transcriptome-metabolome analysis reveals the mechanism of salinity stress mitigation by trehalose-coated silica nanoparticles in lentil. Plant Nano Biol. 214, 100276. doi: 10.1016/j.plana.2026.100276 38826717

[B112] Sarkar M. M. Mukherjee S. Mathur P. Roy S. (2022). Exogenous nano-silicon application improves ion homeostasis, osmolyte accumulation and palliates oxidative stress in Lens culinaris under NaCl stress. Plant Physiol. Biochem. 192, 143–161. doi: 10.1016/j.plaphy.2022.10.001 36242906

[B113] Sharma P. Chaudhary S. Kumar R. (2020). Assessment of biotic and abiotic behaviour of engineered SiO2 nanoparticles for predicting its environmental providence. NanoImpact 17, 100200. doi: 10.1016/j.impact.2019.100200 38826717

[B114] Shi Y. An L. Guo S. Li J. Sun H. Zhang R. . (2025). Nano silicon causes a shift in rhizospheric soil microbial community structure and improves nutrient uptake and assimilation in tomato plants under low temperature. Soil Tillage Res. 248, 106451. doi: 10.1016/j.still.2025.106451 38826717

[B115] Shoukat A. Hossain M. S. Pitann B. Anwar H. Saqib Z. A. Mühling K. H. (2025). Nano zinc and silicon enhance salinity resilience in sugar beet (Beta vulgaris) through improved sodium and chloride compartmentalization and osmotic regulation. J. Agron. Crop Sci. 211, e70129. doi: 10.1111/jac.70129 40046247

[B116] Show S. Tamang A. Chowdhury T. Mandal D. Chattopadhyay B. (2015). Bacterial (BKH1) assisted silica nanoparticles from silica rich substrates: a facile and green approach for biotechnological applications. Colloids Surfaces B. Biointerfaces 126, 245–250. doi: 10.1016/j.colsurfb.2014.12.039 25576815

[B117] Siddiqi K. S. Husen A. Zahra N. Moheman A. (2025). Harnessing silicon nanoparticles and various forms of silicon for enhanced plant growth performance under salinity stress: application and mechanism. Discover Nano 20, 89. doi: 10.1186/s11671-025-04270-2 40439761 PMC12123022

[B118] Siddiqui M. H. Al-Whaibi M. H. (2014). Role of nano-SiO2 in germination of tomato (Lycopersicum esculentum seeds Mill.). Saudi J. Biol. Sci. 21, 13–17. doi: 10.1016/j.sjbs.2013.04.005 24596495 PMC3937468

[B119] Slomberg D. L. Schoenfisch M. H. (2012). Silica nanoparticle phytotoxicity to Arabidopsis thaliana. Environ. Sci. Technol. 18, 10247–10254. doi: 10.1021/es300949f 22889047

[B120] Souri Z. Khanna K. Karimi N. Ahmad P. (2021). Silicon and plants: current knowledge and future prospects. J. Plant Growth Regul. 40, 906–925. doi: 10.1007/s00344-020-10172-7 30311153

[B121] Sun D. Hussain H. I. Yi Z. Siegele R. Cresswell T. Kong L. . (2014). Uptake and cellular distribution, in four plant species, of fluorescently labeled mesoporous silica nanoparticles. Plant Cell Rep. 33, 1389–1402. doi: 10.1007/s00299-014-1624-5 24820127

[B122] Suriyaprabha R. Karunakaran G. Yuvakkumar R. Rajendran V. Kannan N. (2012). Silica nanoparticles for increased silica availability in maize (Zea mays. L) seeds under hydroponic conditions. Curr. Nanosci 8, 902–908. doi: 10.2174/157341312803989033

[B123] Surya K. Sornam G. Asritha M. Paul R. A. I. Easwaran C. Sri B. K. . (2025). Unraveling approaches of silica nanoparticles for next-generation in agricultural sustainability. Front. Sustain. Food Syst. 9, 1677788. doi: 10.3389/fsufs.2025.1677788

[B124] Tian L. Shen J. Sun G. Wang B. Ji R. Zhao L. (2020). Foliar application of SiO2 nanoparticles alters soil metabolite profiles and microbial community composition in the pakchoi (Brassica chinensis L.) rhizosphere grown in contaminated mine soil. Environ. Sci. Technol. 54, 13137–13146. doi: 10.1021/acs.est.0c03767 32954728

[B125] Tiwari A. Sherpa Y. L. Pathak A. P. Singh L. S. Gupta A. Tripathi A. (2019). One-pot green synthesis of highly luminescent silicon nanoparticles using Citrus limon (L.) and their applications in luminescent cell imaging and antimicrobial efficacy. Mater. Today Commun. 19, 62–67. doi: 10.1016/j.mtcomm.2018.12.005 38826717

[B126] Verma K. K. Song X. P. Li D. M. Singh M. Wu J. M. Singh R. K. . (2022). Silicon and soil microorganisms improve rhizospheric soil health with bacterial community, plant growth, performance and yield. Plant Signaling Behav. 17, 2104004. doi: 10.1080/15592324.2022.2104004 35943127 PMC9364706

[B127] Wang T. Long H. Mao S. Jiang Z. Liu Y. He Y. . (2024b). Silicon nanoparticles improve tomato seed germination more effectively than conventional silicon under salt stress via regulating antioxidant system and hormone metabolism. Horticulturae 10, 785. doi: 10.3390/horticulturae10080785 30654563

[B128] Wang S. Xue J. Zhao Y. Du M. Deng L. Xu H. . (2014). Controlled silica deposition on self-assembled peptide nanostructures via varying molecular structures of short amphiphilic peptides. Soft Matter 10, 7623–7629. doi: 10.1039/c4sm01578a 25131511

[B129] Wang P. Zhang H. Hu X. Xu L. An X. Jin T. . (2024a). Comparing the potential of silicon nanoparticles and conventional silicon for salinity stress alleviation in soybean (Glycine max L.): growth and physiological traits and rhizosphere/endophytic bacterial communities. J. Agric. Food. Chem. 72, 10781–10793. doi: 10.1021/acs.jafc.4c00154 38709780

[B130] Wong Po Foo C. Patwardhan S. V. Belton D. J. Kitchel B. Anastasiades D. Huang J. . (2006). Novel nanocomposites from spider silk–silica fusion (chimeric) proteins. Proc. Natl. Acad. Sci. 103, 9428–9433. doi: 10.1073/pnas.0601096103 16769898 PMC1476692

[B131] Xu X. Wan J. Liu G. Lu C. Mao X. Wu J. . (2025). Physiological, transcriptomic and metabolomic analyses reveal the mechanism of CuO and silicon nanoparticles involved in Polygonatum kingianum response to root rot. Chem. Biol. Technol. Agric. 12, 102. doi: 10.1186/s40538-025-00821-y 38164791

[B132] Yadav P. Amir M. Raheem A. Khan S. A. Sharma M. Ansari M. I. (2025). Novel GABA-stabilized gold nanoparticles for plant systems: Synthesis, characterization, and unprecedented effects on growth, physiological function, and nutrient efficiency in Lactuca sativa L. Biocatal Agric. Biotechnol. 67, 103644. doi: 10.1016/j.bcab.2025.103644 38826717

[B133] Yadav A. Bhatia A. Bana R. S. Ranjan R. Dhakar R. Shivay Y. S. . (2026a). Green zinc oxide nanoparticles improve zinc bioavailability and mitigate high temperature stress in rice. Sci. Rep. 18, 2195–2210. doi: 10.1038/s41598-026-36046-8 41611785 PMC12910017

[B134] Yadav V. K. Fulekar M. H. (2019). Green synthesis and characterization of amorphous silica nanoparticles from fly ash. Mater. Today Proc. 18, 4351–4359. doi: 10.1016/j.matpr.2019.07.395 38826717

[B135] Yadav M. George N. Dwibedi V. (2026b). Mycosynthesis of silica nanoparticles from sugarcane bagasse using Aspergillus Niger MSF3: Optimization and economic assessment. Silicon. 18, 2195–2210. doi: 10.1007/s12633-026-03666-8 30311153

[B136] Yamaji N. Mitatni N. Ma J. F. (2008). A transporter regulating silicon distribution in rice shoots. Plant Cell 20, 1381–1389. doi: 10.1105/tpc.108.059311 18515498 PMC2438455

[B137] Yan G. Huang Q. Zhao S. Xu Y. He Y. Nikolic M. . (2024). Silicon nanoparticles in sustainable agriculture: synthesis, absorption, and plant stress alleviation. Front. Plant Sci. 15, 1393458. doi: 10.3389/fpls.2024.1393458 38606077 PMC11006995

[B138] Yi Z. Hussain H. I. Feng C. Sun D. She F. Rookes J. E. . (2015). Functionalized mesoporous silica nanoparticles with redox-responsive short-chain gatekeepers for agrochemical delivery. ACS Appl. Materials Interfaces 7, 9937–9946. doi: 10.1021/acsami.5b02131 25902154

[B139] Yin L. Wang S. Tanaka K. Fujihara S. Itai A. Den X. . (2016). Silicon‐mediated changes in polyamines participate in silicon‐induced salt tolerance in S orghum bicolor L. Plant Cell Environ. 39, 245–258. doi: 10.1111/pce.12521 25753986

[B140] Younes N. A. El-Sherbiny M. Alkharpotly A. A. Sayed O. A. Dawood A. F. Hossain M. A. . (2024). Rice-husks synthesized-silica nanoparticles modulate silicon content, ionic homeostasis, and antioxidants defense under limited irrigation regime in eggplants. Plant Stress 11, 100330. doi: 10.1016/j.stress.2023.100330 38826717

[B141] Younis A. A. Khattab H. Emam M. M. (2020). Impacts of silicon and silicon nanoparticles on leaf ultrastructure and TaPIP1 and TaNIP2 gene expressions in heat stressed wheat seedlings. Biol. Plant 64. doi: 10.32615/bp.2020.030

[B142] Yue L. Wang J. Cao X. Wang C. Ma C. Chen F. . (2023). Silica nanomaterials promote rice tillering and yield by regulating rhizosphere processes, nitrogen uptake, and hormone pathways. ACS Sustain. Chem. Eng 11, 16650–16660. doi: 10.1021/acssuschemeng.3c05419

[B143] Zamani H. Jafari A. Mousavi S. M. Darezereshki E. (2020). Biosynthesis of silica nanoparticle using Saccharomyces cervisiae and its application on enhanced oil recovery. J. Petroleum Sci. Eng. 190, 107002. doi: 10.1016/j.petrol.2020.107002 38826717

[B144] Zhu P. Chen S. Shi Y. (2024). Assessment of nano silicon fertilizer effects on soil nutrients, enzyme activities, and microbial communities. Environ. Sci. Nano 11, 3124–3136. doi: 10.1039/d4en00223g

[B145] Zhu Y. Yin J. Liang Y. Liu J. Jia J. Huo H. . (2019). Transcriptomic dynamics provide an insight into the mechanism for silicon-mediated alleviation of salt stress in cucumber plants. Ecotoxicol Environ. Saf. 174, 245–254. doi: 10.1016/j.ecoenv.2019.02.075 30831473

[B146] Zielonka A. Żymańczyk-Duda E. Brzezińska-Rodak M. Duda M. Grzesiak J. Klimek-Ochab M. (2018). Nanosilica synthesis mediated by Aspergillus parasiticus strain. Fungal Biol. 122, 333–344. doi: 10.1016/j.funbio.2018.02.004 29665959

